# α1,3-fucosylation of MEST promotes invasion potential of cytotrophoblast cells by activating translation initiation

**DOI:** 10.1038/s41419-023-06166-4

**Published:** 2023-10-06

**Authors:** Hao Wang, Xinyuan Cui, Luyao Wang, Ningning Fan, Ming Yu, Huamin Qin, Shuai Liu, Qiu Yan

**Affiliations:** 1 Liaoning Provincial Core Lab of Glycobiology and Glycoengineering, College of Basic Medical Science, Dalian Medical University, Dalian, 116044 China; 2 Department of Pathology, The Second Affiliated Hospital of Dalian Medical University, Dalian, Liaoning 116011 China; 3 Department of Biochemistry and Molecular Biology, Dalian Medical University, Liaoning Provincial Core Lab of Glycobiology and Glycoengineering, Dalian, 116044 China

**Keywords:** Glycobiology, Glycobiology

## Abstract

Embryo implantation into the uterus is the gateway for successful pregnancy. Proper migration and invasion of embryonic trophoblast cells are the key for embryo implantation, and dysfunction causes pregnancy failure. Protein glycosylation plays crucial roles in reproduction. However, it remains unclear whether the glycosylation of trophoblasts is involved in trophoblast migration and invasion processes during embryo implantation failure. By Lectin array, we discovered the decreased α1,3-fucosylation, especially difucosylated Lewis Y (LeY) glycan, in the villus tissues of miscarriage patients when compared with normal pregnancy women. Downregulating LeY biosynthesis by silencing the key enzyme fucosyltransferase IV (FUT4) inhibited migration and invasion ability of trophoblast cells. Using proteomics and translatomics, the specific LeY scaffolding glycoprotein of mesoderm-specific transcript (MEST) with glycosylation site at Asn163 was identified, and its expression enhanced migration and invasion ability of trophoblast cells. The results also provided novel evidence showing that decreased LeY modification on MEST hampered the binding of MEST with translation factor eIF4E2, and inhibited implantation-related gene translation initiation, which caused pregnancy failure. The α1,3-fucosylation of MEST by FUT4 may serve as a new biomarker for evaluating the functional state of pregnancy, and a target for infertility treatment.

## Introduction

The incidence of pregnancy failure is consistently increased [1, 2]. It is estimated that 23 million miscarriages occur every year worldwide [3], and the population prevalence of women who have recurrent pregnancy loss is up to 2.5%, with high probability of infertility. One of the major reasons leading to infertility is embryo dysfunction. Although the molecular causes of pregnancy loss, such as genetic abnormality and pregnancy related gene expression imbalance have been reported, there is still an urgent need to better understand the mechanisms of the embryonic pathogenesis of miscarriage patients, and find the new biomarkers for pregnancy evaluation, diagnosis and therapy.

Trophoblast is a thin layer of cells that helps a mature embryo to attach to the wall of the uterus [4, 5]. Trophoblast directly initiates the apposition, adhesion and attachment of embryo to the receptive uterus [6], and drives embryo to penetrate deeper uterine tissue. It also transforms to vascularization which sets up the communication with maternal blood vessels. Furthermore, trophoblast develops into the functional placenta which supplies nutrients and mediates the gas exchange at the fetal-maternal interface [7]. Thus, trophoblast steers successful implantation, and dysfunction of trophoblast hampers embryo implantation, angiogenesis and placentation, even lead to pregnancy loss and infertility [8]. However, the molecular mechanism associated with trophoblast invasion is not fully clear.

Protein glycosylation is one of the important posttranslational modifications [9]. Glycans carried by a glycoprotein molecule not only affect the biochemical properties of the molecule, but also regulate a myriad of critical physiological and pathological processes, including embryogenesis and implantation [10, 11], immune response [12, 13], host-pathogen recognition [14, 15] and tumorigenesis [16]. Protein fucosylation is an important type of glycosylation, and generally serves as the final step in the biosynthesis of sugar chains. It includes two major forms: N-fucosylation and O-fucosylation. Glycotransferases are responsible for catalyzing the biosynthesis of protein glycosylation. Fucosyltransferases (FUTs) are the key enzymes that transfer the l-Fucose (GDP-Fuc) to the sugar acceptor substrates, yielding fucosylated glycans. Thirteen FUTs (N-FUTs and O-FUTs) have been identified based on the linkage mode of the sugar chain with the peptide. N-FUTs are classified into α1,2-, α1,3/4- and α1,6-FUTs which are responsible for the corresponding α1,2-, α1,3/4- and α1,6-fucosylation epitopes [17–19]. Fucosyltransferase IV (FUT4) catalyzes the α1,3-fucosylation linkage of difucusylated Lewis Y (LeY, Fuc α1–2 Gal-β1–4[Fuc α1–3]GlcNAcβ1) [20].

Accumulating evidence has revealed that the stage-specific protein glycosylation state and its fine regulation by the expression of corresponding glycoenzymes are essential for embryo development and implantation, as well as uterine receptivity in reproduction glycobiology [21–23]. Aberrant protein fucosylation is due to the abnormal expression of FUTs, which is associated with reproductive disorders, including miscarriage. The increased expression level of FUT4 was found in the secretory phase as compared with that in the proliferative phase, and decreased level of FUT4 expression was detected in the serum of infertility patients [24–26]. We previously found that blocking the surface LeY with anti-LeY antibody impaired the outgrowth function of mouse embryos in the implantation window [27]. Lower serum levels of poFUT1 in abortion patients than those in early pregnant women. Reduced poFUT1 also inhibited epithelial-mesenchymal transition (EMT) of trophoblast cells during embryo implantation [23, 28]. Despite the correlations among certain glycoenzymes/glycans in reproduction processes have been studied, the global glycosylation alternations of the early embryonic villous tissues between pregnancy loss and normal pregnancy have not been analyzed. The profound molecular mechanism of protein fucosylation regulated embryo trophoblast cells migration and invasion ability during embryo implantation and pregnancy loss needs further investigation.

Mesoderm-specific transcript (MEST), also named as PEG1, belongs to the alpha/beta hydrolase superfamily. MEST is a single chain glycoprotein composed of 335 amino acids [29]. As a maternal imprint gene, MEST is widely expressed throughout the embryonic developmental period [30]. MEST plays a crucial role in the development of embryo and placenta, as well as fetal growth. Inappropriate MEST expression is linked to the increased probability of early spontaneous miscarriage and severe fetal defects, such as growth abnormality, low birth weight (LBW), or metabolic disorders in human [31]. In addition, Mest-deficient mice exhibited embryonic and placental development disorders. MEST deficiency also causes postnatal growth inhibition, weight gain and multiple organ hypertrophy [32, 33]. Furthermore, decreased expression of MEST has been linked to cancer pathogenesis, such as choriocarcinoma [34], breast cancer [31], lung adenocarcinomas [35] and lymphoblastoid [36], etc. MEST is an essential determinative molecule in embryo development and maturation. Although MEST has three isoforms produced by alternative splicing, the full-length protein has only one N-glycosylation site at Asn163. Whether MEST presents fucosylation modification and how does the fucosylation regulates the functions of MEST in embryo implantation are worthwhile to explore.

Using Lectin array analysis, we compared the trophoblast glycosylation state in miscarriage patients with that in early normal pregnancy control. Results showed that α1,3-linkage fucose glycan, especially LeY, was significantly decreased on the trophoblast of miscarriage patients. Applying proteomics and translatomics, we identified MEST as one of the differentially expressed scaffold proteins of LeY. We further found that the α1,3-fucosylation of MEST mediates its binding with the eukaryotic initiation factor eIF4E2, and initiates the embryo implantation-related gene translation. This study provides a new insight into the function and mechanism of protein fucosylation of trophoblast during normal embryo implantation and miscarriage.

## Results

### Decreased α1,3-fucosylation/FUT4/LeY in the villous tissues of miscarriage patients

**Fig. 1:**
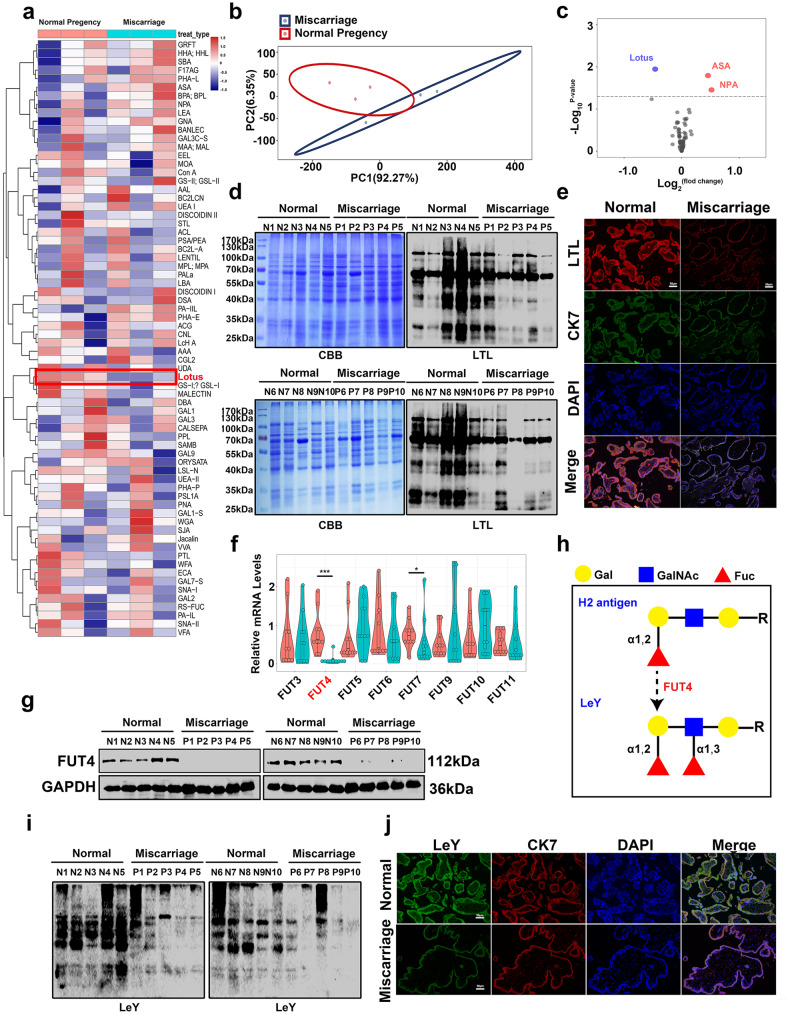
Expression of α1,3-fucosylation, α1,3-fucosyltransferases (FUTs) and LeY in trophoblast from early pregnant women and miscarriage patients. **a** Heatmap showing the expression level of all differentially glycans. **b** The value was normalized by z-score. **c** Volcano plots showing the DEGs through pairwise comparison. Numerals indicate the number of genes upregulated (red) or downregulated (blue). **d** Lectin blot analysis showed the α1,3-linkage fucose on trophoblast of early pregnancy women and abortion patients. **e** Immunofluorescent staining of Lotus Tetragonolobus Lectin (LTL) on embryonic trophoblast tissues. Detection of FUT4 expression by real-time PCR (**f**) and western blot (**g**). **h** Diagram of FUT4 catalyzed the LeY biosynthesis. Detection of LeY level by western blot (**i**) and Immunofluorescence staining (**j**). *p < 0.05, ***p < 0.001.

To reveal the global glycosylation traits in the embryonic trophoblast, villous tissues from early normal pregnancy women and miscarriage patients were collected for Lectin array analysis. Differential levels of glycosylation were exhibited (Fig. 1a–c). Among the 70 types of lectins which recognize and bind the specific sugar chain epitopes, the Lotus Tetragonolobus Lectin (LTL) binding α1,3-fucosylated glycans in the trophoblast from miscarriage patients was significantly decreased (*p* < 0.01), compared with that in normal control (Fig. 1c). The changes of LTL glycans was further confirmed by detecting the α1,3-fucosylation level using Lectin blot (Fig. 1d). The immunofluorescent staining also revealed that the α1,3-fucosylation was mainly located in the embryonic trophoblast, and the staining was weaker in the miscarriage samples compared with normal control. CK7 was used as the positive marker of trophoblast epithelium (Fig. 1e).

We then compared the expression levels of α1,3-FUTs(FUT3–7, 9–11) in the trophoblast of normal pregnancy and miscarriage patients. Among these α1,3 FUTs, FUT4 was decreased significantly in the trophoblast tissues of miscarriage patients, as examined by Real-time PCR and western blot (Fig. 1f, g). LeY is a difucosylated oligosaccharide which contains α1,2- and α1,3-linkage fucose. FUT4 is the key enzyme responsible for last step of LeY biosynthesis by adding UDP-Fuc to the GlcNAc β1 of H2 antigen precursor through α1,3-fucosylation [37, 38] (Fig. 1h). Therefore, we further analyzed the LeY level, the representative α1,3-linkage fucosylation in trophoblast from different tissue samples. Western blot results and immunofluorescent staining showed that the level of LeY was lessen in miscarriage trophoblast tissues (Fig. 1i, j). The flow cytometry data also confirmed that LeY level was decreased in miscarriage patients (Supplementary Fig. 1). In addition, similar alteration of α1,3-fucosylation, FUT4 and LeY in serum were also detected in the pregnancy women and miscarriage patients (Supplementary Fig. 2a, b). All these findings suggest that the overall level of protein α1,3-fucosylation (fucosylated glycans, key enzyme FUT4, especially difucosylated LeY oligosaccharide) was reduced in trophoblast from miscarriage patients.

### Silencing FUT4 inhibits LeY biosynthesis, and impairs the potential of trophoblast cells proliferation, migration and invasion

**Fig. 2:**
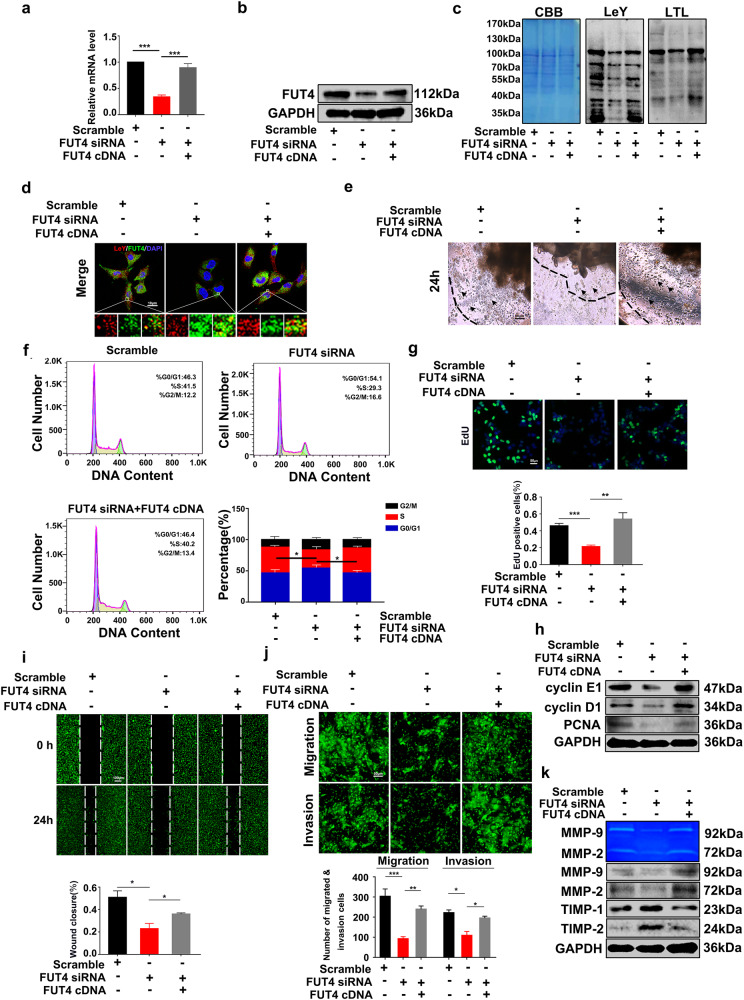
FUT4 siRNA inhibits LeY biosynthesis, further impairs trophoblast cells proliferation, migration and invasion potential. Real-time PCR and western blot analysis of FUT4 at the mRNA level (**a**) and protein level (**b**). **c** Lectin blot assay and western blot analysis of a1,3-fucosylation and LeY on HTR-8/SVneo cells after transfected with scramble-siRNA, FUT4 siRNA, or co-transfection with FUT4 siRNA and poFUT4 cDNA. **d** Immunofluorescence staining of LeY and FUT4 on HTR-8/SVneo cells. Lower panel is magnification of up panel of white frame. **e** Extravillous explants from 7 weeks of gestation maintained in culture on matrigel were transfected with scramble RNA, FUT4 siRNA and FUT4 cDNA, representative pictures of explants were taken after 24 h of culture in vitro. **f** The cell cycle arrest phases were assessed by Flow cytometry in HTR-8/SVneo cells. **g** Edu assay evaluated cell proliferation potential. **h** Western blot detected the change of cyclin E1, D1 and PCNA. **i** Cell scratch test detected migration ability of HTR-8/SVneo cells. **j** Matrigel transwell assay detected cell invading ability of HTR-8/SVneo cells. **k** Gelatin zymography assay and western blot detected the activation and alteration of MMPs and TIMP1/2. *p < 0.05, ***p < 0.001.

Silencing FUT4 by specific siRNA significantly inhibited FUT4 expression at both mRNA and protein levels, whereas transfecting FUT4 cDNA partially reversed the inhibitory effects of FUT4 siRNA in trophoblast HTR-8/SVneo cells (Fig. 2a, b). Consistently, FUT4 siRNA inhibited the global α1,3-fucosylation determined by LTL Lectin blot, and the LeY biosynthesis was decreased as well. FUT4 cDNA transfection partially alleviated the inhibitory effect of FUT4 siRNA on the expression of both α1,3-fucosylation and LeY as examined by Lectin blot, western blot (Fig. 2c) and immunofluorescent staining (Fig. 2d). To evaluate the impact of the FUT4 and LeY expression on trophoblast invasion potential, extravillous explants from first-trimester woman villus tissues were transfected with FUT4 siRNA or co-transfected with FUT4 siRNA and cDNA and plated on the Matrigel-coated dishes. The explants anchored on Matrigel started to outgrowth after 24 h in culture. The results showed that less cells and lowered motility in FUT4 siRNA group, while the FUT4 cDNA restored the extension (Fig. 2e). Consistently, Flow cytometry (FCM) analysis of cell cycle showed that FUT4 siRNA decreased G1/S phase transition, whereas FUT4 cDNA transfection partially restored the inhibition (Fig. 2f). Similar change was observed in the 5-ethynyl-2′-deoxyuridine (EdU) DNA integration detection of the proliferative cells (Fig. 2g). Cell cycle proteins (cyclin E1 and cyclin D1) and proliferation cell nuclear antigen (PCNA) were downregulated in FUT4 siRNA treatment group. These were reversed by FUT4 cDNA transfection (Fig. 2h). In addition, FUT4 siRNA significantly decreased the trophoblast cells migration and invasion ability. Oppositely, FUT4 cDNA transfection relieved the inhibition as examined by Wound-healing assay and transwell assay (Fig. 2i, j), as well as MMP-2 enzymatic activity determined by gelatin zymography (Fig. 2k). These results suggest that downregulation of FUT4 inhibits LeY biosynthesis and impairs trophoblast proliferation, migration and invasion potential.

### Identification of LeY scaffolding glycoproteins

**Fig. 3:**
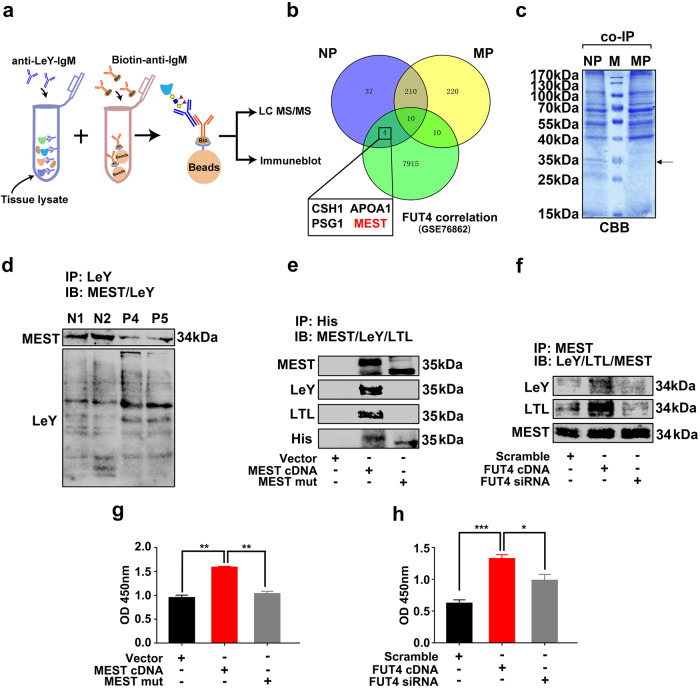
Global search LeY modified glycoproteins in trophoblast from early pregnancy women and miscarriage patients. **a** Schematic of the LeY modified glycoproteins identification procedure. The anti-LeY antibody coupled with LeY scaffold glycoproteins was purified, and the resulting proteins were subjected to liquid chromatography-tandem mass spectrometry (LC–MS/MS) based proteomics or immune blot. **b** Venn diagrams showing the number of LeY modified glycoproteins in trophoblast of normal early pregnancy women and abortion patients, and FUT4 correlated proteins in abortion patients (GSE76862). **c** Pull down proteins separated with SDS-PAGE and staining with CBB. CBB: Coomassie brilliant blue. **d** Immunoprecipitation (IP) and western blot analysis of LeY on MEST of trophoblast of normal early pregnancy women (N1, N2) and abortion patients (P4, P5). **e** IP and western blot analysis of α1,3 fucosylation and LeY on MEST of HTR cell after transfected with his-tagged MEST cDNA and his-tagged MEST N163Q mutate plasmid. **f** IP and western blot analysis of α1,3 fucosylation and LeY on MEST of HTR cells after transfected with FUT4 cDNA or FUT4 siRNA. **g**, **h** ELISA assay analysis of LeY on MEST of HTR cells after transfected with FUT4 cDNA or FUT4 siRNA. *p < 0.05, **p < 0.01 and ***p < 0.001.

To explore the specific LeY-carrier or scaffolding glycoproteins in embryonic tissues, anti-LeY antibody (IgM) pull down assay (co-IP) was employed (Fig. 3a). Using s, a total of 489 LeY-containing glycoproteins were identified. Among them, a total of 261 LeY-containing proteins were found in normal pregnancy women (NP) group, and 450 LeY-containing proteins were identified in miscarriage patients (MP) group. There were 41 LeY-containing proteins identified only in NP group. According to the Gene Expression Omnibus (GEO) (GSE76862), 7939 mRNAs were correlated with FUT4 on the human trophoblast (Cor > 0.7, *p* < 0.05). Combined with our results, 4 candidate glycoproteins (CSH1, APOA1, PSG1, MEST) found in NP group were associated with FUT4 expression (Fig. 3b). The gene ontology (GO) analysis further assessed the biofunctions of candidate glycoproteins (Supplementary Table 1), and identified that MEST was the candidate glycoproteins participated in embryo development. Coomassie Brilliant blue staining (CBB) also showed lighter staining of the 35 kDa MEST in MP group, which also consist with LC–MS/MS results (Fig. 3c). The immunocoprecipitation (IP) with anti-LeY antibody and immunoblotting further confirmed MEST contained LeY modification which was decreased in the villous trophoblastic tissues from MP than NP (Fig. 3d).

According to NetNGlyc predication, MEST is a glycoprotein which contains only one N-glycosylation modification site at Asn163. We subsequently tested whether MEST was modified by LeY at Asn163. The his-tagged MEST cDNA plasmid and MEST N163Q mutant plasmid (MEST mut) were constructed to detect LeY level on MEST in HTR-8/SVneo cells. IP results showed that MEST contained LeY and α1,3-fucosylation modification, while MEST mut group exhibited neither LeY nor α1,3-fucosylation, which confirmed that MEST scaffolding LeY oligosaccharide modification at Asn163 (Fig. 3e). Furthermore, we found that FUT4 cDNA transfection increased the biosynthesis of LeY and α1,3-fucosylation on MEST; whereas FUT4 siRNA decreased LeY and α1,3-fucosylation biosynthesis (Fig. 3f). Lectin-ELISA assay also showed the similar change of LeY level on MEST (Fig. 3g, h). These results provide the evidence that MEST contains LeY oligosaccharide modification which is regulated by FUT4.

Then, we illustrated the functions of LeY on MEST on embryonic trophoblast cells proliferation, migration and invasion ability. Firstly, both MEST cDNA and MEST mut plasmid upregulated MEST expression in gene and protein level. However, the lower molecular weight MEST was detected in the MEST mut group, compared with the normal molecular weight MEST in cDNA group, indicating that MEST contains N-glycosylation modification, and was consisted with our previous results (Supplementary Fig. 3a–c). Furthermore, MEST cDNA promoted cell proliferation, migration and invasion ability, whereas MEST mut abated the promotive function (Supplementary Fig. 3d–i).

### LeY on MEST mediates the interaction of MEST and eIF4E2

**Fig. 4:**
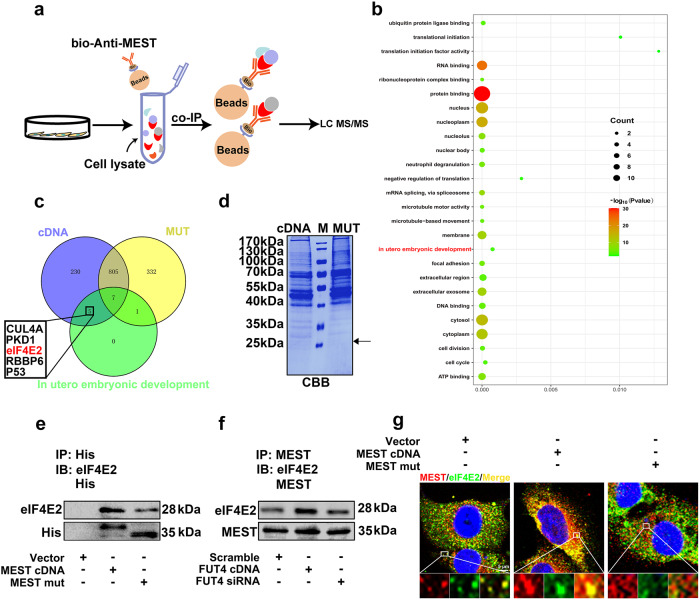
α1,3 fucosylated-MEST mediate interaction of MEST and eIF4E2. **a** Schematic of analysis strategy for identification MEST interacting protein. **b** GO analysis of the proteins interacted with MEST. **c** Venn diagrams showing the number of the proteins interact with MEST form NP, MP and GO analysis-based function. **d** Pull down proteins separated with SDS-PAGE and staining with CBB. **e** N-glycosylation of MEST at Asn163 enhances the interaction of MEST and eIF4E2. HTR-8/SVneo cells transfected with His-tagged MEST cDNA and His-tagged MEST N163Q mutant plasmid (MEST mut), respectively, then co-immunoprecipitated (co-IP) with anti His antibody and blotted with anti-eIF4E2. **f** FUT4 regulated interaction between MEST and eIF4E2 by regulating LeY on MEST. HTR-8/SVneo cells transfected with FUT4 cDNA and FUT4 siRNA, respectively, then interaction was detected by co-IP assay. **g** Confocal immunofluorescence showing the location of MEST and eIF4E2 in cells, lower panel is magnification of up panel of white frame.

To address the mechanism of LeY modified MEST on cytotrophoblast cells functions, the proteins which interacted with MEST were screened using co-immunoprecipitation (co-IP) and LC–MS/MS assay. The workflow of the strategy was illustrated as Fig. 4a. HTR-8/SVneo cells transfected with MEST cDNA or MEST mut were lysed and immunopurified with anti-MEST antibody, followed by analysis with LC–MS/MS. Proteomics analysis identified 1380 proteins which interacted with MEST directly or indirectly. These proteins were involved in the biological processes, including embryonic development and cell division by GO enrichment analysis (Fig. 4b). Among these, 1047 proteins were exclusive in NP group, whereas 1145 proteins were identified in MP group. The differentially expressed five candidate proteins (CUL4A, PKD1, eIF4E2, RBBP6 and P53) involved in embryo development were selected for further investigation (Fig. 4c). Among these candidate proteins, eIF4E2 is a translational repressor that mediates target mRNA translation initiation. Considering the translation reprogramming is an important step during embryo implantation. Here, we selected eIF4E2 for further study.

Employing co-IP, blotting assay and confocal immunofluorescent assay, we subsequently confirmed the interaction of MEST and eIF4E2. The proteins were co-immunoprecipited with His-tagged antibody from the lysate of cells with transfected MEST cDNA or MEST mut plasmid. The MEST stably interacted with eIF4E2; whereas this interaction was reduced in MEST mut group, suggesting that LeY on MEST determined the interaction of MEST and eIF4E2 (Fig. 4e). CBB staining also showed lighter staining in MEST mut group at 28 kDa, which is consistent with the result of co-IP and LC–MS/MS (Fig. 4d). Transfection of FUT4 cDNA increased LeY biosynthesis and promoted the binding of MEST with eIF4E2. In contrast, FUT4 siRNA transfection significantly inhibited the interaction of these two molecules by decreasing LeY biosynthesis (Fig. 4f). Moreover, confocal observation showed that MEST and eIF4E2 were well co-located in the cytoplasm of trophoblast HTR-8/SVneo cells, which further confirmed the interaction of MEST was interacted with eIF4E2 (Fig. 4g). In summary, LeY on MEST plays key roles mediating MEST and eIF4E2 interaction.

### LeY on MEST preferentially stimulates the translation initiation and expression of embryo implantation-related genes

**Fig. 5:**
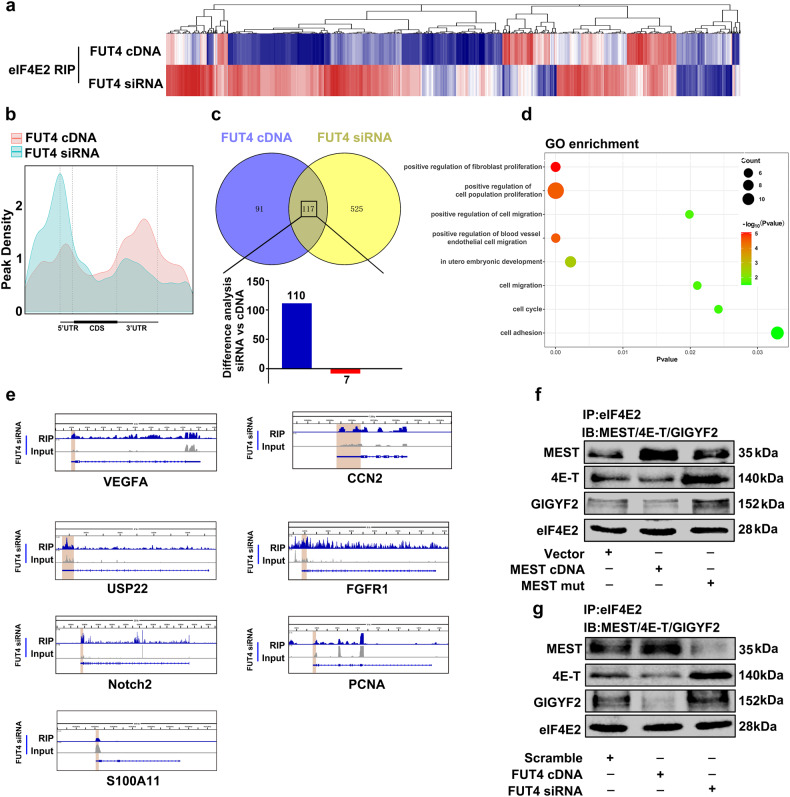
Genome-wide α1,3 fucosylated MEST preferentially stimulates the translation and expression of implantation relate genes. **a** Heatmap showing the alternation of mRNAs immunoprecipitated by anti-eIF4E2 antibody in HTR-8/SVneo cells using RIP-seq assay. HTR-8/SVneo cells transfected with FUT4 siRNA or FUT4 cDNA. **b** Binding peak density analysis of binding site of mRNA and eIF4E2. **c** Venn diagrams displays the number of genes in FUT4 cDNA group (blue), FUT siRNA group (yellow), or both (overlapping) group. The changed of 117 overlapping mRNAs is shown in column graphs. **d** GO analysis of the mRNAs with high affinity to eIF4E2. **e** Integrative Genomics Viewer (IGV) tracks showing the binding of eIF4E2 at the 5’-UTR regions of the representative genes. **f** The eIF4E2-MEST/4E-T/GLGYF2 interaction is decreased in HTR-8/SVneo cells transfected with MEST mut, compared with MEST cDNA transfection. eIF4E2 co-IP was performed, and the immunoprecipitated fractions were analyzed by immunoblotting for the indicated proteins. **g** The eIF4E2-MEST/4E-T/GLGYF2 interaction in HTR-8/SVneo cells transfected with FUT4 cDNA or FUT4 siRNA, compared with scramble group. eIF4E2 co-IP was performed, and the immunoprecipitated fractions were analyzed by immunoblotting for the indicated proteins.

It is reported that eIF4E2, a homolog of eIF4E, represses the translation initiation by binding the cap structure of target mRNA. We hypothesized that MEST without α1,3-fucosylation modification could alter the translation process, especially during reproduction. We firstly identified the global alternations of mRNA translation, using an RNA immunoprecipitation followed by high-throughput sequencing (RIP-seq) assay. The HTR-8/SVneo cells transfected with FUT4 cDNA or siRNA, then mRNA samples were IP with anti-eIF4E2 antibody. A total 733 mRNAs were identified (Fig. 5a). The binding of eIF4E2 and 5′-UTR suppressed the target gene translation. Binding peak density analysis revealed that mRNA peaks were enriched at the 5′-UTR in FUT4 siRNA group; whereas the peaks mainly located at 3′-UTR in FUT4 cDNA group, suggesting FUT4 siRNA inhibited gene translation by facilitating eIF4E2 binding to mRNA (Fig. 5b). Among the identified mRNAs, 208 mRNAs bound to eIF4E2 in FUT4 cDNA transfection group, and 642 mRNAs in FUT4 siRNA transfection cells. Among these, 525 mRNAs bound to eIF4E2 restrict in FUT4 siRNA transfection group, 117 mRNAs emerged on both FUT4 cDNA and FUT4 siRNA group. Among the 117 mRNAs, 110 mRNAs exhibited higher level of binding to eIF4E2 in FUT4 siRNA transfection group, compared with FUT4 cDNA group (Fig. 5a, c). For the high level of mRNAs in FUT4 siRNA group, GO analysis showed a significant enrichment for genes associated with cell proliferation, migration, cell cycle, cell adhesion and embryonic development (Fig. 5d). Furthermore, seven embryo implantation-related mRNAs (VEGFA, CCN2, USP22, GFGR1, Notch2, PCNA and S100A11) were selected to verify the binding of eIF4E2 and mRNAs. Integrative genomics viewer (IGV) analysis displayed the binding of eIF4E2, and mRNAs peaks were located in 5′-UTR in those seven mRNAs (Fig. 5e).

eIF4E2 is mainly responsible for the formation of a translation repressor complex with 4E-T and/or GIGYF2 to inhibit mRNA translation initiation. Furthermore, we assessed the role of α1,3 fucosylated-MEST on eIF4E2/4E-T/GIGYF2 complex formation. As shown in Fig. 5f, MEST N163Q mutation significantly inhibited the binding of MEST and eIF4E2, whereas facilitated the interaction of eIF4E2 and 4E-T/GIGYF2, compared with MEST cDNA group. In addition, FUT4 cDNA transfection facilitated the interaction between eIF4E2 and MEST, and inhibited the binding of eIF4E2 and 4E-T/GIGYF2. FUT4 siRNA played the oppositely function to FUT4 cDNA (Fig. 5g). These results indicate that LeY on MEST plays crucial role during the competitive combination of eIF4E2 to METS or 4E-T/GIGYF2, by which to further influence the target gene translation initiation.

### α1,3-fucosylation of MEST at Asn163 preferentially stimulates the translation and expression of embryo implantation-related genes

**Fig. 6:**
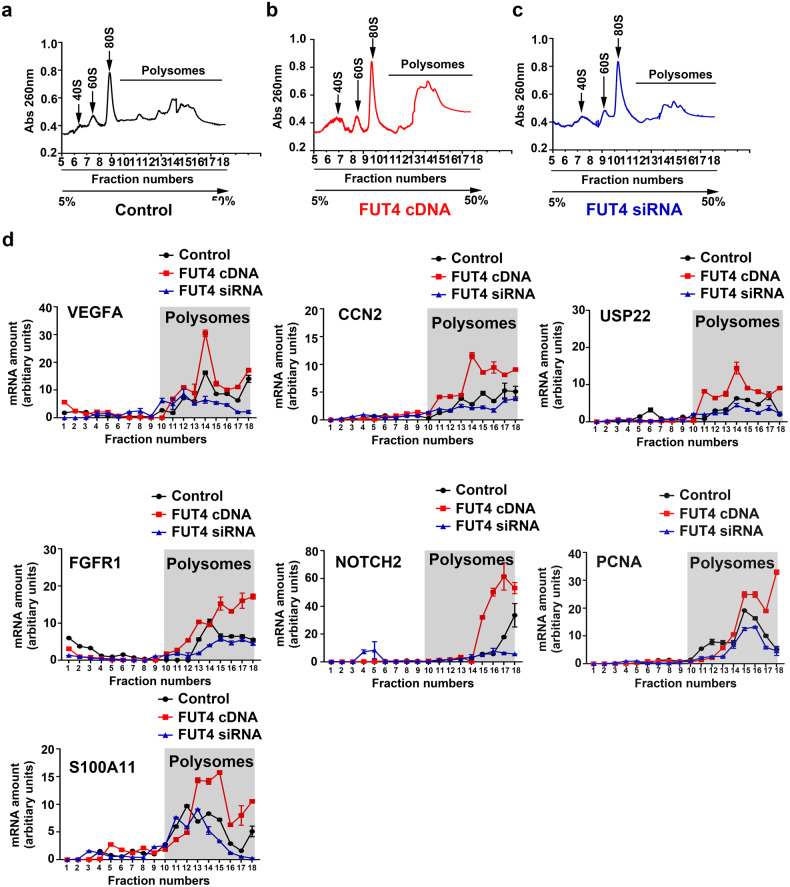
α1,3 fucosylated MEST at Asn163 preferentially stimulates the translation and expression of implantation relate genes. Polysome profiling of control (**a**), FUT4 cDNA (**b**) and FUT siRNA (**c**) transfected HTR-8/SVneo cells. **d** Real-time PCR measuring the abundance of indicated genes mRNA in each fraction.

The mRNAs translation levels of above mentioned seven selected genes were detected by polysome profiling and Real-time PCR analysis. Results showed that FUT4 cDNA transfection increased, but FUT4 siRNA decreased the polysomal fraction in HTR-8/SVneo cells, suggesting increased translation efficiency in FUT4 cDNA transfection group and decreased translation efficiency in FUT4 siRNA treated group compared with control group (Fig. 6a–c). In addition, the distributions of selected mRNAs (VEGFA, CCN2, USP22, FGFR1, NOTCH2, PCNA, S100A11 and GAPDH) in pooled poly-tomonoribosomal reactions were detected. Results showed that FUT4 cDNA transfection significantly accelerated these mRNAs translation effect, whereas FUT4 siRNA treatment suppressed mRNA translation effect in polysomal fraction (Fig. 6d). This evidence suggests that LeY on MEST at Asn163 has the positive effect on translation initiation of specific embryo implantation-related genes.

### Silencing FUT4 and anti-LeY antibody blockage inhibits embryo implantation in vivo

**Fig. 7:**
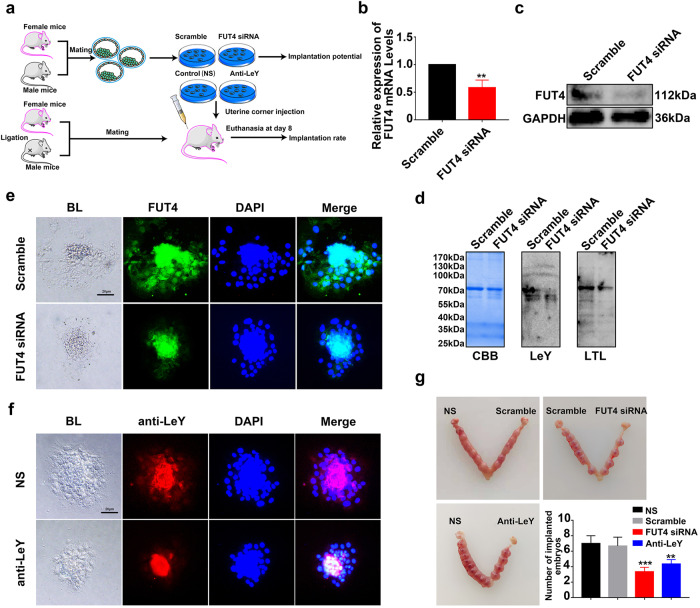
Silencing FUT4 and anti-LeY antibody blockage inhibit embryo implantation in vivo. **a** Schematic of strategy for mouse model in vivo. **b**, **c** Real-time PCR and Western blot analysis of embryonic FUT4 expression after transfected with FUT4 siRNA. **d** Western blot and Lectin blot detect LeY and α1,3 fucosylation level. **e**, **f** Embryo transfected with scramble RNA, FUT4 siRNA, or treated with normal saline (NS) or anti-LeY antibody, respectively, pictures were taken after 24 h of culture in vitro. **g** Numbers of implantation embryos in the uterus on day 8 pregnancy. The statistical analysis was shown. **p < 0.01, ***p < 0.001.

To address the biological significance of FUT4 and LeY in embryo implantation in vivo, mouse blastocysts were collected from pregnant mouse on day 3. After the blastocysts were treated with FUT4 siRNA or anti-LeY antibody, one group of blastocysts were used for examining embryo outgrowth to evaluate implantation potential, and another group of blastocysts were transplanted into the pseudopregnancy mice to evaluate embryo implantation rate (Fig. 7a). For in vitro cultured embryo, FUT4 siRNA significantly inhibited FUT4 and LeY biosynthesis as determined by Real-time PCR and western blot analysis (Fig. 7b–d). In addition, the cultured embryo exhibited impaired outgrowth ability in numbers and the extension of trophoblast cells in FUT4 siRNA group, compared with scramble group (Fig. 7e). Meanwhile, anti-LeY antibody treated embryo displayed similar change with FUT4 siRNA group (Fig. 7f). Embryo cultured medium was collected to detect the activity of MMPs by gelatin zymography. FUT4 siRNA and anti-LeY antibody blockage significantly reduced enzymatic activity of MMP-2 and MMP-9 in cultures, compared with scramble and normal saline control group (Supplementary Fig. 4a, b). The embryo implantation rate analysis on day 8 revealed that a smaller number of implanted embryos in FUT4 siRNA group than scramble group (10 ± 1 vs. 4 ± 1). The decreased implantation rate was observed in anti-LeY antibody group as well (10 ± 1 vs. 4 ± 1) (Fig. 7g). Correspondingly, FUT4 siRNA significantly inhibited the expression of the molecules, including cyclin D1, NOTCH2, PCNA and VEGFA, in the embryo after different treatments (Supplementary Fig. 4c). These results demonstrate that FUT4 siRNA inhibited LeY biosynthesis, which led to decreased embryo implantation-related mRNAs translation, and further hampered embryo implantation.

## Discussion

**Fig. 8:**
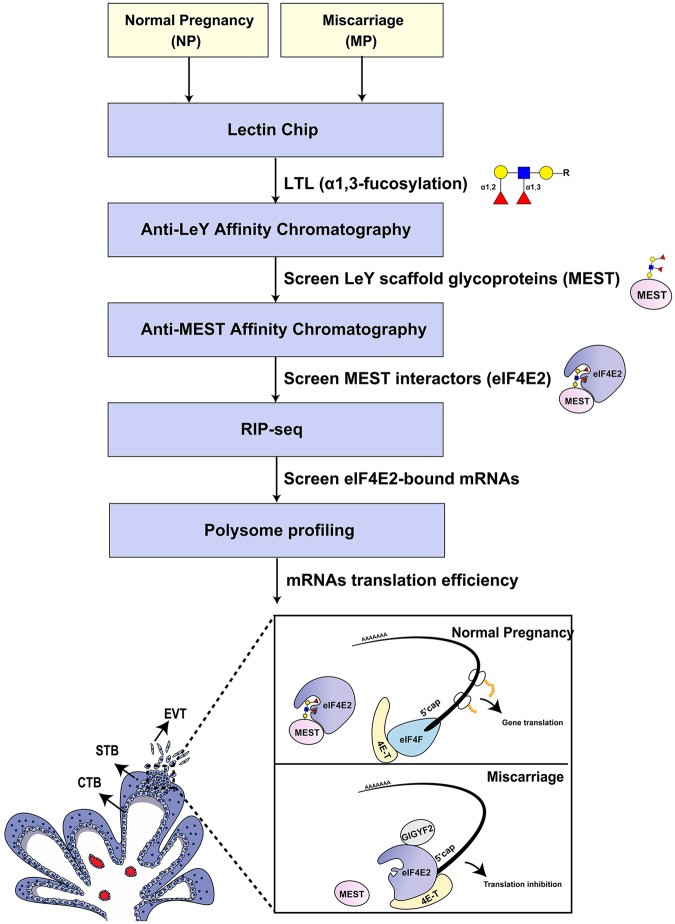
A summary scheme of LeY on MEST promotes embryonic trophoblast cells migration and invasion by activating translation initiation. The α1,3-fucosylation of glycoproteins in the villi of normal pregnancy and miscarriage was screened by Lectin Chip. Anti-LeY affinity chromatography was used to screen Ley scaffold glycoproteins by anti-LeY antibody. The target MEST was selected, and the specific interaction protein (eIF4E2) was discovered by anti-MEST affinity chromatography. By RIP-seq and polysome profiling, implantation-related mRNAs bound with eIF4E2 were confirmed. Reduced LeY on MEST inhibited binding of eIF4E2 with MEST, migration and invasion of trophoblast cells by inactivating translation initiation.

Systemic analysis of the protein glycosylation (glycans/glycoenzymes) helps to identify the general distinct variations in physiological reproduction processes and pathological pregnancy diseases. Here, we systematically analyzed the glycosylation alterations in normal and abnormal early pregnancy from the embryonic aspect. By Lectin array based glycomics analysis, we first found that decreased α1,3-fucosylation, especially difucosylated Lewis Y (LeY) glycan, in the villus trophoblast of miscarriage patients compared with normal pregnancy women. Using trophoblast cells and villous explants, we also found that the downregulated LeY by silencing FUT4 inhibited the trophoblast cells proliferation, migration and invasion potential. Furthermore, silenced LeY modification on MEST dramatically hampered MEST binding with translation factor eIF4E2, and inhibited implantation-related gene translation initiation, which caused pregnancy failure (Fig. 8). The results suggest that general protein fucosylation may serve as a potential glycobiological biomarker for trophoblast function evaluation and clinical miscarriage prediction and diagnosis.

Accumulative data of glycobiology reveal that the alternations of the specific glycosyltransferase usually give rise to the changes the glycans biosynthesis, and thus influences the functions of the glycans [39–41]. Fucosyltransferases are mainly responsible for fucosylated oligosaccharide biosynthesis, which are involved in many physiological and pathological processes. For example, the overexpression FUT1 increased the α1,2-fucosylation, drove cancer stemness in hepatocellular carcinoma [42]. The FUT8 catalyzed core fucosylation of N-glycans, and knockdown of FUT8 rescued the immunosuppressive function in triple negative breast cancer cells [43], whereas upregulation of FUT8 was identified as features of metastatic melanoma [44, 45]. poFUT1 promoted endometrial decidualization and uterine angiogenesis by increasing O-fucosylation of human endometrial stromal cells [28, 46]. Furthermore, we found that LeY biosynthesis was changed by regulating the expression of FUT4 with FUT4 cDNA or siRNA in HTR-8/SVneo trophoblast cells. The deletion of LeY further leads to trophoblast cell dysfunction. Our studies suggest that lower FUT4 may hampers trophoblast proliferation, migration and invasion ability, which is one of the critical causes for miscarriage, by decreasing LeY biosynthesis.

Glycosylation, especially fucosylation, is the fine regulator in determining the biochemical properties and the functions of a scaffold protein, such as in protein folding, targeted delivery, signal transduction, transcription and translation processes in reproduction. Studies have demonstrated that LeY on EGFR determinate the dimerization and phosphorylation of EGFR, and mediated EGFR signal pathway activation [47, 48]. O-fucose glycans of Notch was essential for Notch signaling pathway to involve in the regulation of embryo development [49, 50]. Although studies have revealed that fucosylated specific scaffold proteins were related to the biological process in embryo implantation, the landscape of fucosylation substrates in human trophoblast of normal pregnancy and miscarriage is still largely unknown. Here, we identified 491 different fucosylated proteins in villi tissues between miscarriage and normal pregnancy. We further identified four LeY modified proteins (CSH1, APOA1, PSG1 and MEST), and found that MEST contained α1,3-linkage fucose, especially LeY oligosaccharide at Asn163. We also provided evidence that the α1,3-fucosylation, especially LeY expression on MEST determined MEST positive function during process of trophoblast cells proliferation, migration and invasion.

To further explore the mechanism of MEST glycosylation-mediated function, co-IP and proteomics were employed. We initially evaluated the role of fucosylation of MEST on its interactions with other proteins and discovered that MEST interacted with eIF4E2 had a strong reaction with MEST. The expression of LeY on MEST played crucial role for the binding of MEST and eIF4E2, indicating that MEST fucosylation is associated with the translation in reproduction.

Translational reprogramming is one of the important events that occurs during embryo implantation [51]. Proliferation and implantation-related genes exhibit high translation activity, whereas apoptosis-related genes display lower translation activity during embryo implantation. Interestingly, increasing lines of evidence confirm that glycosylation is involved in translation, which indicates the participation of the glycosylation in translational reprogramming [9]. It was reported that O-GlcNAcylation of RACK1 increased oncogenes translation potential [52]. However, little is known about fucosylation in translation process, especially during embryo implantation period. During mRNA translation initiation, a heterotrimeric complex, eukaryotic initiation complex factor 4F (eIF4F), including eIF4E1, eIF4A and eIF4G, is recruited to the 5’ cap of mRNA to activate translation process. eIF4E2 is a negative regulator of translation by binding to the 5’ cap of mRNA in the translation initiation [53]. There are several factors associated with eIF4E during the translation initiation regulation. One of important factors is eIF4E-Transporter (4E-T) [54]. The 4E-T protein directly binds to eIF4E1 through binding YX4LL (Y30TKEELL) motif and facilitate eIF4E1 binding to eIF4G, forming initiation complex. eIF4E2 also binds to 4E-T, but it does not associate with eIF4G and cap structure, thus it acts as a translational repressor by competing with eIF4E1. Beyond 4E-T, Grb10-interacting GYF protein 2 (GIGYF2) is another cofactor accomplishing with eIF4E2 in translational repressor complex [55]. eIF4E2 and GIGYF2 are required for the proper embryonic development as the disruption of these genes causes perinatal lethality in mice [56, 57]. By RIP-seq analysis, we screened the eIF4E2 binding mRNAs after manipulating the expression of FUT4 with its siRNA or cDNA transfection. Our results suggest that FUT4 facilitates MEST binding to eIF4E2 through increasing LeY biosynthesis on MEST, by which to positively control the translation in trophoblast cells migration and invasion and embryo implantation.

In summary, we applied glycomics, proteomics combined with translatomics to provide the insights into mechanisms of miscarriage. We revealed that decreased α1,3-fucosylation, especially LeY oligosaccharide and FUT4 on the trophoblast of miscarriage patients in comparison with early normal pregnancy women. In addition, downregulating LeY by silencing FUT4 inhibited trophoblast proliferation, migration and invasion potential. Moreover, we provided novel evidence showing that reducing LeY carried on MEST hampers the binding of MEST and eIF4E2 and inhibits implantation-related gene translation, which leads to pregnancy failure. The general α1,3-fucosylation state, especially LeY and FUT4 may serve as the potential new diagnostic biomarkers and therapeutic targets for miscarriage patients.

## Materials and methods

### Human placental villi tissue collection

All experimental protocols for human study were in accordance with the approved guidelines by the Institutional Review Boards of Dalian Medical University (2021104). Tissue samples of normal and abortion women at the age of 25–35 were collected from The Secondary Affiliated Hospital of Dalian Medical University (Dalian, China) between 2020 and 2021. The pregnant (20 cases) and miscarriage women were (20 cases) confirmed by ultrasound detection at 6–10 gestational weeks. Normal human placental villi obtained from normal women undergoing legal abortion for nonmedical reasons. The miscarriage tissue samples were from the first trimester of patients who undergone induced abortion. The arraryCGH detection was negative and the serum progesterone level was less than 30 ng/ml for these patients. And all samples were used for immunohistochemical fluorescent staining, protein and mRNA extraction.

### Cell culture

The human embryonic HTR-8/SVneo was purchased from the American Type Culture Collection (Manassas, VA, USA). HTR-8/SVneo cells were cultured in DMEM/F12 supplemented with 10% FBS, 100 µg/mL streptomycin and 100 U/mL penicillin. Cells were cultured at 37 °C under 5% CO_2_ in humidified air incubator. The medium was changed every 2 days. The cell lines used in this study were authenticated by short tandem repeat (STR) profiling.

### Cell transfection

HTR-8/SVneo cells were seeded onto 60-mm culture dishes and then transfected with FUT4 cDNA, siRNA (sense: 5′-GUU UGG AUG AAC UUC GAG UTT-3′, anti-sense: 5′-ACUCGAAGUUCAUCCAAACTT-3′), MEST CDNA, MEST mut, which were purchased from Genepharma (Shanghai, China), using lipofectamine 2000 following the manufacturer’s instructions. After 48 h, RNA and protein samples were collected.

### RNA isolation and real-time quantitative PCR

Total RNA from cells and tissues were treated with Trizol reagent (Invitrogen). A PrimeScript RT Reagent Kit (TaKaRa, Dalian, China) was used to synthesize cDNA. Real-time PCR was performed with SYBR Premix Ex Taq (TaKaRa) and the reactions were performed using ABI Prism 7500 Detection system (Applied Biosystem, Foster City, CA, USA). GAPDH was used as loading controls and the relative quantity was calculated using the 2^−∆∆CT^ method. All of them were obtained from Genepharma. Primer sequences were as followings: FUT4: 5′-AAG GTC CAG GCC CAC TGA AG -3′(forward), 5′-CAG TTC AGG TGA CAG AGG CTC AA-3′(reverse); PCNA: 5′-TAA AGA AGA GGA GGC GGT AA-3′ (forward), 5′-TAA GTG TCC CAT GTC AGC AA-3′(reverse); Cyclin D1: 5′-TGT CCT ACT ACC GCC TCA CA-3′ (forward), 5′-CTT GGG GTC CAT GTT CTG CT-3′ (reverse); Cyclin E1: 5′-TGC AGC CAA ACT TGA GGA AA TC-3′ (forward), 5′-TAG TCA GGG GAC TTA AAC GC CA-3′ (reverse); GAPDH: 5′-GCA CCG TCA AGG CTG AGA AC-3′ (forward), 5′-TGG TGA AGA CGC CAG TGGA-3′ (reverse). Each experiment was repeated at least three times.

### Western and Lectin blot

Tissues were lysed in RIPA lysis buffer including protease inhibitor cocktail (Roche, Basel, Switzerland). Cells were lysed in loading buffer containing protease inhibitor cocktail. Equal proteins were loaded onto 12% SDS-PAGE gels and electrotransferred onto nitrocellulose membranes (Merck Millipore, Billerica, MA, USA). After blocking in 5% non-fat dry milk for 2 h, the membranes were incubated with the primary antibody at 4 °C overnight: FUT4 (19497-1-AP), PCNA (10205-2-AP), Cyclin D1 (26939-1-AP), Cyclin E1 (11554-1-AP), MMP-2 (10373-2-AP), MMP-9 (10375-2-AP), TIMP1 (16644-1-AP), TIMP2 (17353-1-AP), MEST (11118-1-AP), eIF4E2 (12227-1-AP), GIGYF2 (24790-1-AP) and GAPDH (10494-1-AP) (Proteintech, Wuhan, China); 4E-T (sc-134233, Santa Cruz, USA); LeY (ab-3359, Abcam, USA); biotinylated LTL (B-1325-2, Vector Laboratories, California, USA). After washing, the membranes were incubated with HRP-labeled goat anti-rabbit IgG (SA00001-2), HRP- HRP-labeled streptavidin (SA00001-0) or labeled goat anti-mouse IgG (SA00001-1) (Proteintech, Wuhan, China) for 1 h. The visualize immunoreactivity bands were detected with ECL detection system (Bio-Rad, USA).

### Immunofluorescent and Lectin fluorescent staining

Paraffin-embedded slices (tissues) were prepared, followed by deparaffinization and rehydration. After antigen being exposed, 0.3% H_2_O_2_ was used to remove endogenous peroxidases by incubation for 30 min and blocking with goat serum for 30 min at room temperature to block nonspecific binding. Or coverslips (cells) were fixed in 4% paraformaldehyde for 30 min, followed by blocking with 1% goat serum for 2 h. Primary antibodies and Lectin: LTL (1:500), LeY (1:200) and Ck7 (1:300) (15539-1-AP, Proteintech, Wuhan, China) antibody were incubated overnight at 4 °C. After washing, Alexa Fluor^TM^ 594-TRITC conjugated goat anti-rabbit IgG (1:100) (A-11012, Invitrogen), Alexa Fluor^TM^ 488-FITC-conjugated goat anti-mouse IgG (1:100) (A-11008, Invitrogen) and Alexa Fluor^TM^ 488-FITC (A32360, Invitrogen) or Alexa Fluor^TM^ 594-TRITC (S11227, Invitrogen)‐conjugated streptavidin were incubated for 45 min at room temperature following treatment with DAPI (1:1000) for 3 min. Finally, anti-fade solution (Beyotime) was added to mount the coverslips or slices and photographed under the fluorescent microscope (Olympus).

### Isolation of trophoblast and flow cytometry

For normal and miscarriage women, complete first-trimester placental villi were delicately divided into smaller segments. These segments underwent a sequence of three enzymatic digestion cycles using an evenly blended combination of TrypLE (Gibco, Catalog 12604021) and Accumax (STEM CELL, Catalog 07921). This enzymatic digestion process was carried out for a duration of 20 minutes at a temperature of 37 °C. The collected cell suspensions underwent filtration through a 70 mm mesh filter. Trophoblast cells were then subjected to immunomagnetic purification using the EasySep FITC selection kit (STEM CELL, Catalog 17682) along with a FITC-conjugated anti-ITGA6 antibody (Invitrogen, Catalog 11049582, 1:200). Collected trophoblast cells were incubated for LeY (Abcam, Mouse monoclonal IgM, Catalog ab3359, 1:200) antibody, and secondary antibody was Goat Anti-Mouse lgM/PE (Solarbio, Catalog K0055G-PE, 1:200). The expression of LeY was detected in trophoblast cells through flow cytometry.

### Wound-healing assay

Cells transfected the indicated plasmids were seeded in six-well plates, and a plastic pipette was used to create an artificial wound. Images of injury area were taken with an inverted microscope. The average extent of the wound closure for each group was quantified.

### Immunoprecipitation

Immunoprecipitation was performed with protein G agarose beads (Thermo Fisher Scientific, Waltham, MA, USA) according to the standard procedures. Briefly, cells after transfection were incubated in IP lysis buffer for 30 min at room temperature. The extracts were incubated with anti-LeY-antibody (2 μg/mL), and the protein G agarose beads were incubated with goat anti-mouse IgM (62-6820, Invitrogen) (1:100) at 4 °C overnight. The beads were washed for three times with washing buffer and the immunoprecipitants were purified by protein G agarose beads with gentle rocking for 3 h. Finally, the beads were washing for five times with washing buffer and added in 50 μl SDS-loading buffer. The whole cell lysates were incubated at 70 °C for 15 min followed with western blot analysis.

### 5-Ethynyl-2′-deoxyuridine (EdU) incorporation assay

EdU (5-ethynyl-2′-deoxyuridine) provided in the kit is a nucleoside analog of thymidine and is incorporated into DNA during active DNA synthesis. Rapid detection of cellular DNA replication activity by specific EdU-based reaction with Apollo® fluorescent dyes. Cells (3 × 10^4^) transfected the indicated plasmids were seeded in 24-well plates and treated with 10 µM EdU (Ribobio EdU Cell Proliferation Kit with Alexa Fluor 488) used according to the manufacturer’s instructions was added to the cell culture and the cells were incubated for an additional 2 h at 37 °C. Every plate was fixed with 4% paraformaldehyde at room temperature for 30 min. And add glycine (2 mg/mL) on a shaking bed for 5 min. Next, permeabilized with 0.3% Triton X-100 for 10 min, then Apollo solution was added, and the cells were incubated on a shaking bed for 30 min at room temperature in the dark. The cell nucleus was incubated with Hoechst 33342 for 15 min at room temperature. Images were captured by the fluorescent microscope (Olympus). Data are presented as the ratio of the fluorescent positive cells to total cells. Three separate experiments were repeated.

### Flow cytometric analysis

HTR-8/SVneo cells were seeded onto 60-mm culture dishes and then transfected with FUT4 cDNA, siRNA, MEST CDNA, MEST mut, which were purchased from Genepharma. And cells were washed PBS, then digested to a single cell suspension with 0.25% trypsin-EDTA solution for 2 min at 37 °C. Next, Cells were collected by centrifuging at 800 × *g* for 5 min at room temperature. After resuspension, cells were fixed with chilled 75% alcohol at 4 °C for overnight. Cells were washed with cold PBS for 3 times, stained with propidium iodide (PI) (50 µg/mL) and RNase A (100 µg/mL) for 1 h at 37 °C in the dark. A BD FACSAria™ Fusion was used to obtain cell cycle data and analyzed by FLOWJO V10. Three separate experiments were repeated.

### Matrigel cell invasion and migration assay

For matrigel invasion assay, transwell inserts (6.5 mm, Costar) were precoated with 35 µL of 1 mg/mL matrigel matrix (Becton Dickinson). For migration assay, the transwell inserts were not precoated with matrigel. The medium with 10% FBS was added to the lower chamber, whereas cells in serum-free medium were added to the upper chamber. After cultured for 16 h, the cells were removed by cotton swab on the upper side of the transwell inserts. The bottom of the cells was fixed in methanol and stained with crystal violet. The number of invaded or migrated cells was taken with an inverted microscope (Olympus) in five random fields. Three separate experiments were repeated.

### Gelatin zymography assay

The conditioned media were prepared without boiling or reduction and used as the samples to detect matrix metalloproteinase (MMP)-2/9 activities. The samples were electrophoresed on 10% SDS-PAGE gels with 1% gelatin. After electrophoresis, the gels were washed with 2.5% Triton X-100 for 1 h and incubated in 50 mM Tris-Cl, pH 7.8, and 5 mM CaCl_2_ (18 h, 37 ˚C). Finally, 0.1% Coomassie blue R250 stained the gels and destained in 10% acetic acid and 10% methanol in H_2_O.

### Extravillous explants culture

The explant culture was performed as described previously. In brief, tips of first trimester placental villi (7 weeks) were collected from women and stored in 4 °C sterilized normal saline. After that, villi were dissected into small tissue sections (2–3 mm) and explanted in Millicell-CM culture dish inserts (0.4 mm pore size, Millipore, Carrigtwohill, Co. Cork) precoated with phenol red-free matrigel substrate (Becton Dickinson, Bedford, MA). The dissected tissue pieces were put on top of each drop and allowed anchorage, then were supplemented with 10% FBS and DMEM/F12 medium. Villi successfully anchored on Matrigel matrix and initiated outgrowth and were treated with FUT4 cDNA, siRNA or vehicle alone. EVT sprouting and migration from the distal end of the villous tips were recorded daily for up to 2 days. The extent of migration was taken with an inverted microscope (Olympus). Three separate experiments were repeated.

### Co-Immunoprecipitation and LC–MS/MS analysis

The normal pregnancy and miscarriage pregnancy tissues were harvested and lysed in RIPA buffer on ice for 30 min. Tissues lysate was centrifuged at 12,000 rpm for 10 min at 4 °C, the supernatant was firstly precleared by incubating with biotinylated IgM antibody, and streptavidin beads were incubated with Bioin-IgM for overnight on rotating device (DR-Mix, HEROS). HTR-8/SVneo cells were harvested and lysed in RIPA buffer on ice for 30 min. Cell lysate was centrifuged at 12,000 rpm for 20 min at 4 °C, the supernatant was used for subsequent experiments. The MEST antibody was added to 40 μl of Protein A/G PLUS-Agarose (sc2003, Santa Cruz) and incubated 3 h at room temperature on rotating device. The supernatant and beads were incubated for 3 h on rotating device. Immunoprecipitated proteins were collected by centrifugation at 2500 rpm for 5 min at 4 °C. Beads was rinsed with PBS for 5 times. Fifty microliters of elution buffer (25 mM Tris HCl pH 7.5, 1% SDS completed with protein inhibitor cocktail) was added to the tips. The supernatant was separated by SDS-PAGE, and the gal (a lane) was digested by trypsin for LC–MS/MS.

The samples were first disassociated by proteases, and then were reconstituted with mobile phase A (2% ACN, 0.1% FA), centrifuged at 20,000 × *g* for 10 min, and the supernatant was taken for injection. Separation was carried out by a Shimadzu LC-20AD model nanoliter liquid chromatograph. The sample was first enriched in the trap column and desalted, and then entered a tandem self-packed C18 column (75 micron internal diameter, 3 micron column size, 15 cm column length), and separated at a flow rate of 300 nl/min by the following effective gradient: 0–6 min, 6% mobile phase B (98% ACN, 0.1% FA); 6–40 min, mobile phase B linearly increased from 6% to 25%; 40–48 min, mobile phase B rose from 25% to 40%; 48–51 min, mobile phase B rose from 40% to 90%; 51–55 min, 90% mobile phase B; 55.5-60 min, 6% mobile phase B. The nanoliter liquid phase separation end was directly connected to the mass spectrometer. The peptides separated by liquid phase chromatography were ionized by a nanoESI source and then passed to a tandem mass spectrometer LTQ Orbitrap Velos (Thermo Fisher Scientific, San Jose, CA) for DDA (data-dependent acquisition) mode detection. The protein identification used experimental MS/MS data and aligned with theoretical MS/MS data from database to obtain results. The whole process started from converting raw MS data into a peak list and then searching matches in the database.

### RNA-immunoprecipitation (RIP) analysis

RIP assay was performed using the EZ-Magna RIP RNA-Binding Protein Immunoprecipitation Kit (Cat. #17-701, Millipore, USA). Cells were cultured in 100 cm^2^ dishes to 60–70% confluence, then transfected with FUT4 cDNA and siRNA. Then, cells were harvested by scraping. One RIP reaction required 100 μl of cell lysate from 1.0 × 10^7^ cells. Next, 5 μg of purified antibodies and corresponding IgG was added to the 100-μl cell lysate, and the mixture was incubated with rotation overnight at 4 °C. Antibody eIF4E2 (12227-1-AP, Proteintech), and normal rabbit IgG (PP64B, Millipore) were used for RIP assay. Normal mouse immunoglobulin G (IgG) (Millipore) was regarded as negative control. Samples were incubated with Proteinase K buffer, and immunoprecipitated RNA was isolated. The RNA concentration was measured by a Nanodrop (Thermo Scientific). Finally, Samples are sent for sequencing.

### Polysome fractionation

HTR-8/SVneo cells were seeded onto 100 mm culture dishes and then transfected with FUT4 cDNA and siRNA purchased from Genepharma. After that, cells were washed with cycloheximide containing phosphate-buffered saline before being lysed using polysome lysis buffer (10 mM Tris-Cl, pH 7.4, 5 mM MgCl_2_, 100 mM KCl, 1% (v/v) Triton X-100, 0.5% (w/v) deoxycholate in RNase-free water, supplemented with protease/phosphatase inhibitor cocktail, 2 mM dithiothreitol, 1000 U/mL RNasin and 100 μg/mL cycloheximide) for 10 min on ice. Post-nuclear fractions were obtained by centrifuging the lysates at 16,000 × *g* for 15 min at 4 °C. Fifty optical density units of lysate were layered on a 10–60% sucrose gradient and spun for 5 h at 36,000 × *g* and 4 °C. Then, fractions were obtained using fractionator (Biocomp Instruments, Fredericton, Canada). RNA was isolated from individual fractions using TRIzol (Invitrogen) and subject to either real-time PCR.

### Detection of glycosylated MEST by Lectin-ELISA

To detect the glycosylated MEST, the Lectin-ELISA method was first developed and optimized. The primary antibody was coated in 96-well plate at 4 °C overnight. After brief wash with 0.5 % Triton-PBS (TPBS, pH 7.6), the cell lysates (100 µL, 1 µg/mL) from HTR-8/SVneo trophoblast cells after different treatment were added to the well and incubated at 4 °C overnight. And the samples were washed with washing buffer for three times. And blocking with BSA to prevent nonspecific binding for 1 h at 37 °C. Then, followed by adding biotin-labeled LTL (1:300) or LeY (1:200) and incubated for 2 h at 37 °C. After wash with washing buffer, add 50 µL of substrate reagent for coloring and incubated for 15 min. Then, add 50 µL of stop solution. The optical density value was read out at 450 nm on spectrophotometer (Multiskan Ascent, Thermo Electron Corporation, USA). Three separate experiments were repeated.

### Mouse embryos collection and transfer

All animal experiments from this study were approved by the Animal Ethics Committee of Dalian Medical University (AEE21045). Moreover, the experimental processes were in accordance with the Experimental Animal Management Regulations of Dalian Medical University. The species of Kunming mice (6-8 weeks) were from the Laboratory Animal Center of Dalian Medical University, China. 50 female mice and 15 male mice were ordered for this experiment. The female mice were randomly divided into two groups: a non-pseudopregnancy group (*n* = 35) and a pseudopregnancy group (*n* = 15). The male mice were randomly divided into two groups: a normal group (*n* = 10) and a ligation group (*n* = 5). Mice were stabled under controlled conditions (temperature 20–25 °C; humidity 60%). The mice with vaginal plugs in the next morning, which was defined as the first day of pregnancy. On the day 3.5, the pregnant mice were euthanized by cervical dislocation, and the uteri were collected. Mouse embryos were flushed from the uterus with warm PBS. Next, embryos were placed in 96-well plates, and cultured at 37 °C under 5% CO_2_ in humidified air according to the standard procedures, followed by transfection with FUT4 siRNA. The attachment and spreading status (48 h) were observed under the microscope. Pseudopregnant female mice were used for embryo transfer were prepared by naturally mating with vasectomized males obtained. The semen secretions produced by sterile males were necessary for the uterus to receive embryos for transfer. To obtain a recipient, two females (7–12 weeks) of age were placed with a vasectomized male in the afternoon. The pregnant mice were confirmed by vaginal plugs in the next morning. On days 2.5–3, differently treated mouse morula or blastocysts were transferred to the bilateral uterus of pseudopregnant female mice. The uterus was removed from the recipient 8 days after embryo transfer.

### Statistical analysis

GraphPad Prism® (GraphPad Software Inc) was used for statistical analysis. Data were presented as mean ± SD unless otherwise indicated from 3 independent experiments. For the analysis of the difference between groups, two‐tailed unpaired t test or one‐way ANOVA was performed. The statistical significance was indicated as the follows: *p < 0.05, **p < 0.01 and ***p < 0.001.

### Supplementary information

Authorship Change Approval

supplementary fig and table

Original Data File WB

## Data Availability

Data supporting the present study are available from the corresponding author upon reasonable request.
