# Supplementary material for: α1,3-fucosylation of MEST promotes invasion potential of cytotrophoblast cells by activating translation initiation
Source: Cell Death Dis. 2023 Oct 6;14(10):651. doi: 10.1038/s41419-023-06166-4 (PMC10556033; doi:10.1038/s41419-023-06166-4)

Hao Wang, Xinyuan Cui, Luyao Wang, Ningning Fan, Ming Yu, Huamin Qin, Shuai Liu, Qiu Yan.

All the co-authors agree to the add and in order of authors.

Hao Wang,


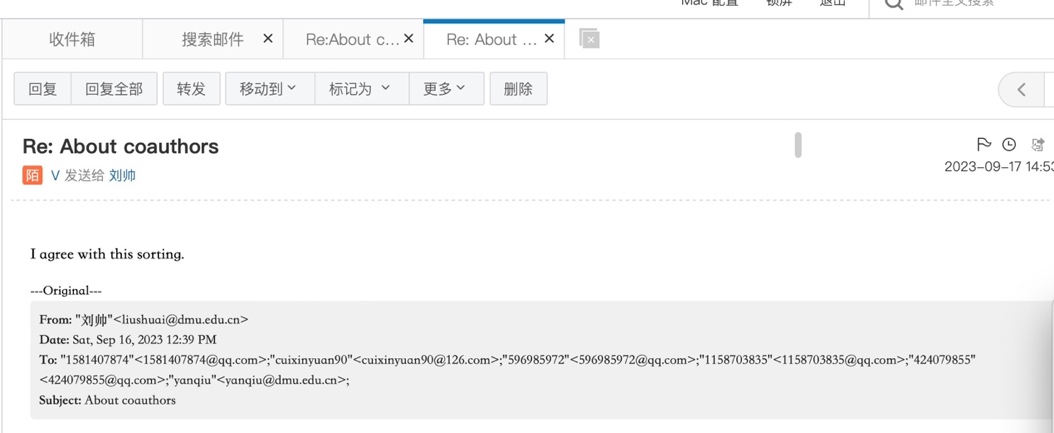


Xinyuan Cui,


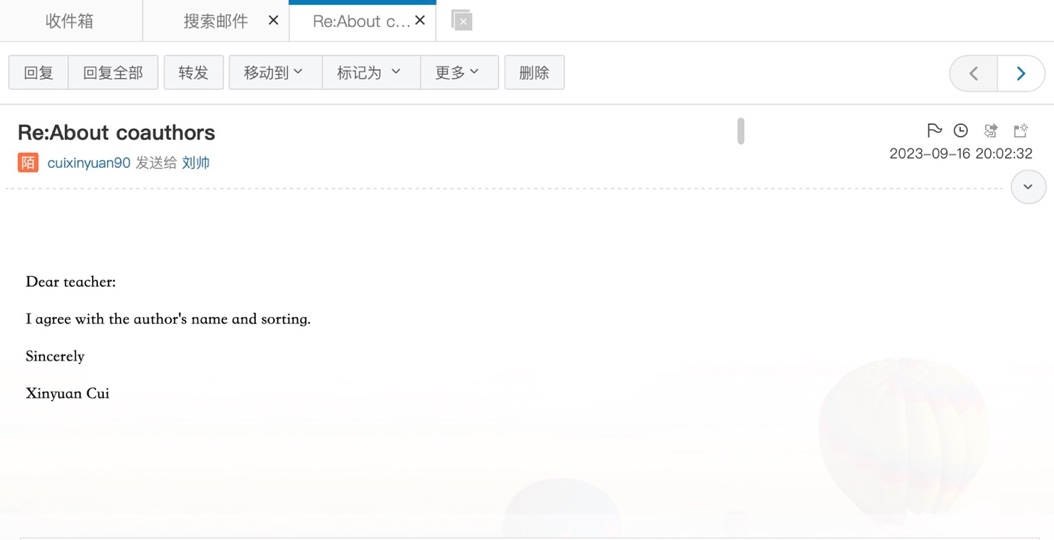


Luyao Wang,


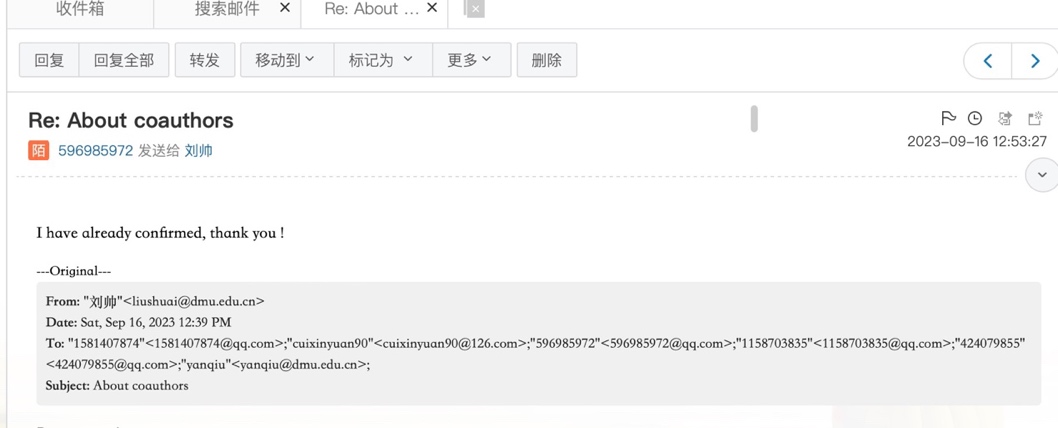


Ningning Fan,


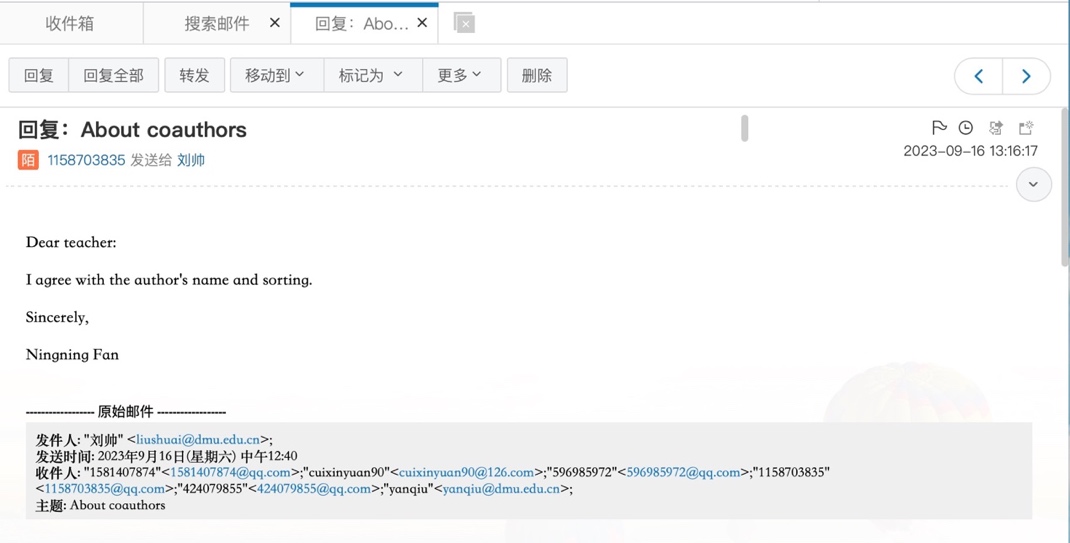


Ming Yu,


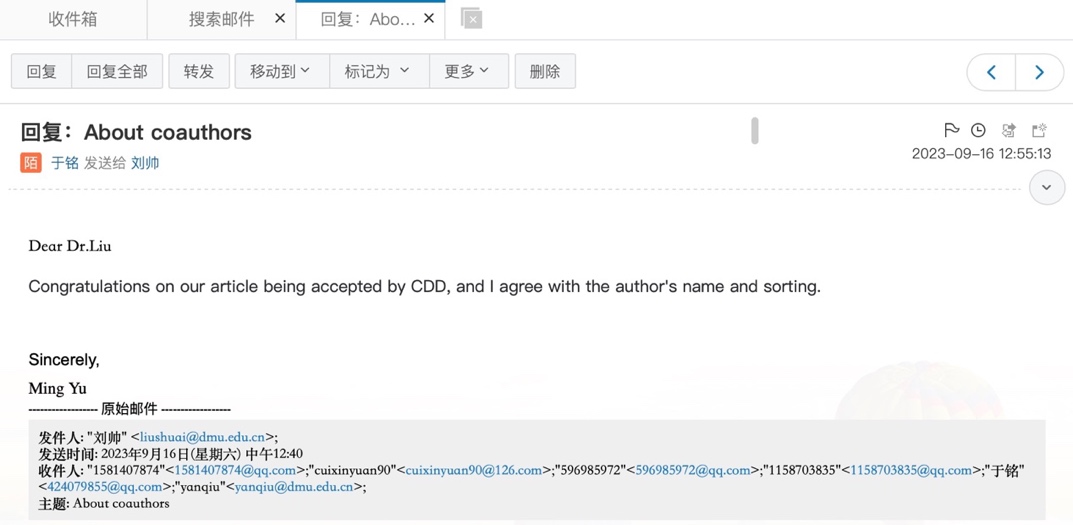


Huamin Qin,


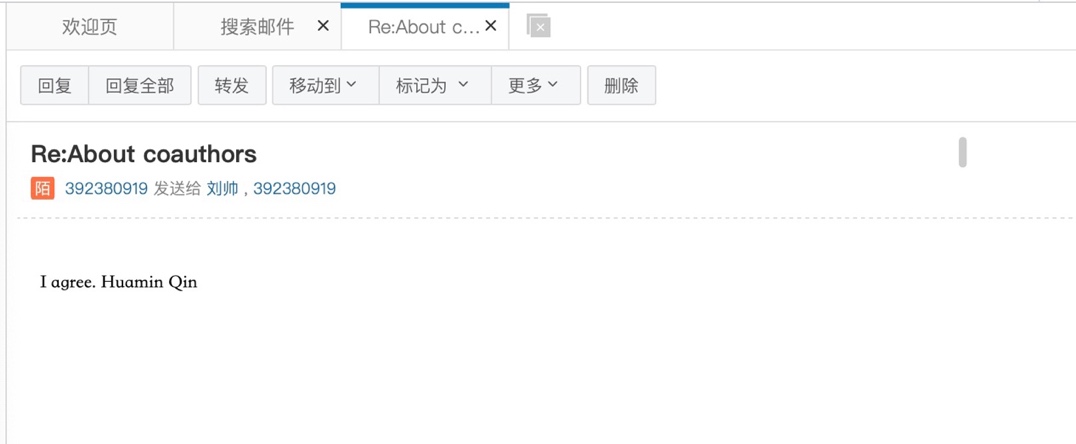


Qiu Yan


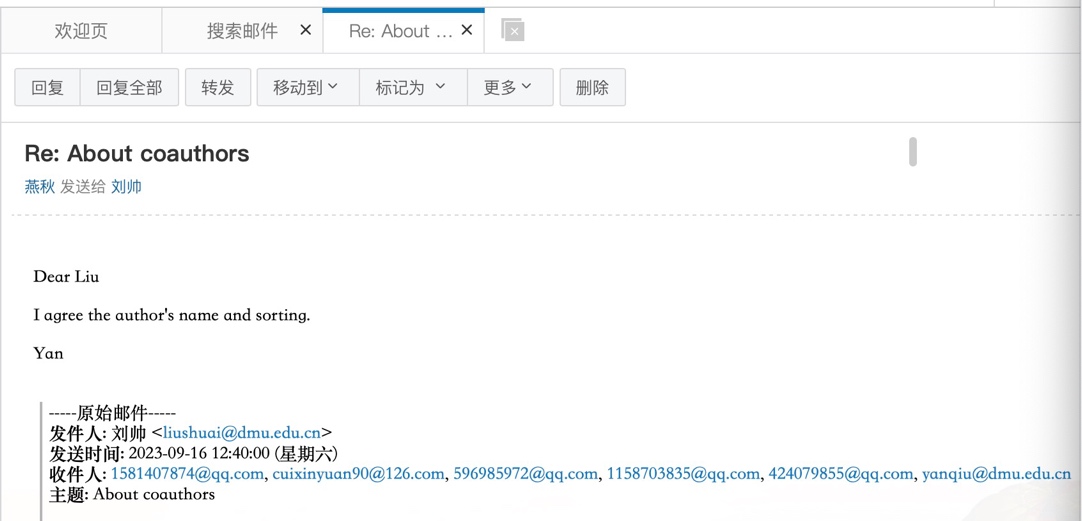


Liu Shuai sent email to coauthors


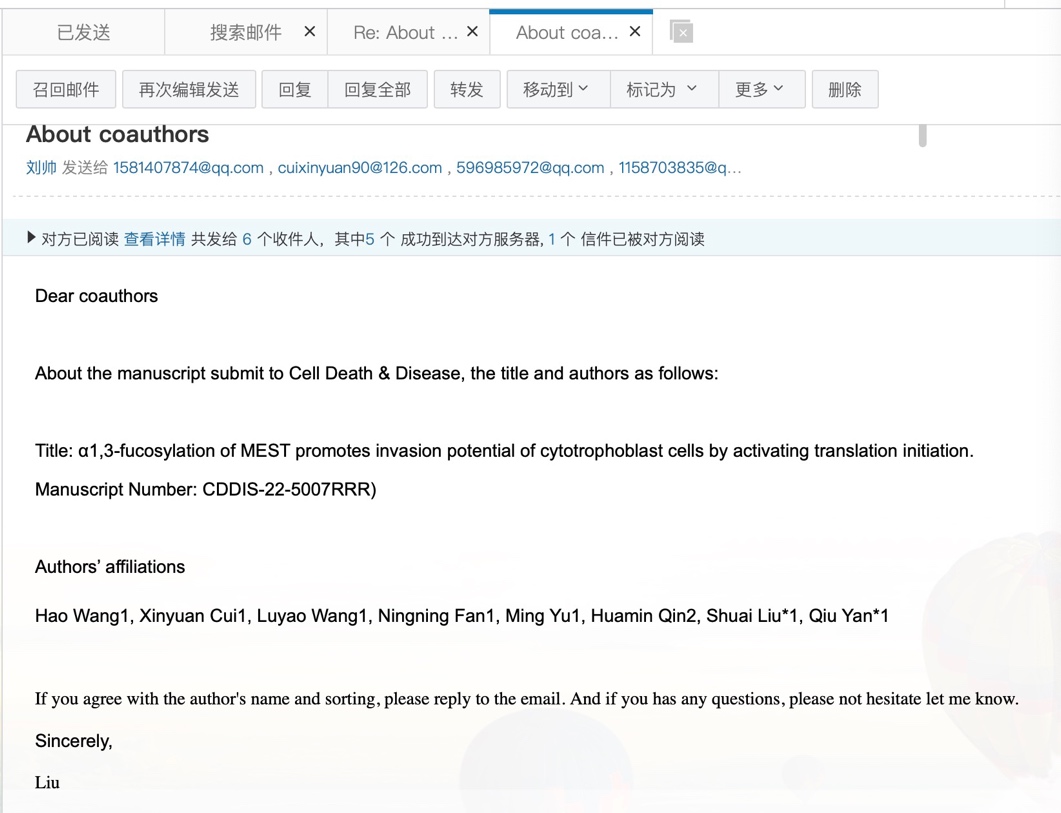

Supplement: Supplementary file 1 — Authorship Change Approval [file 41419_2023_6166_MOESM1_ESM.docx]
