# Supplementary material for: α1,3-fucosylation of MEST promotes invasion potential of cytotrophoblast cells by activating translation initiation
Source: Cell Death Dis. 2023 Oct 6;14(10):651. doi: 10.1038/s41419-023-06166-4 (PMC10556033; doi:10.1038/s41419-023-06166-4)
Supplement: Supplementary file 2 — supplementary fig and table [file 41419_2023_6166_MOESM2_ESM.pdf]

Supplementary

Supplementary Figure 1

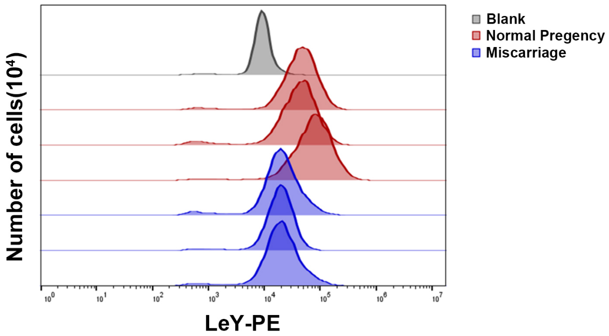

Supplementary Figure 1. Flow cytometry histograms of LeY level on cytotrophoblast cells from early pregnancy women and miscarriage patients.

Supplementary Figure 2

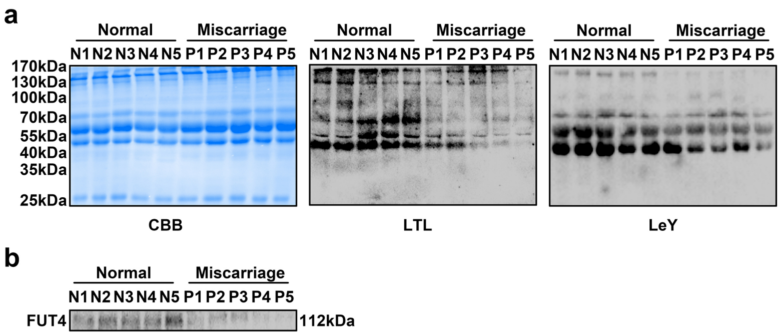

Supplementary Figure 2. Levels of α1,3-fucosylation, LeY and FUT4 in serum from early pregnant women and miscarriage patients. (a) Lectin blot and western blot analysis showed the α1,3-linkage fucose and LeY levels in serum from early pregnancy women and abortion patients. **(b)** Detection of FUT4 expression Western blot in serum.

Supplementary Figure 3

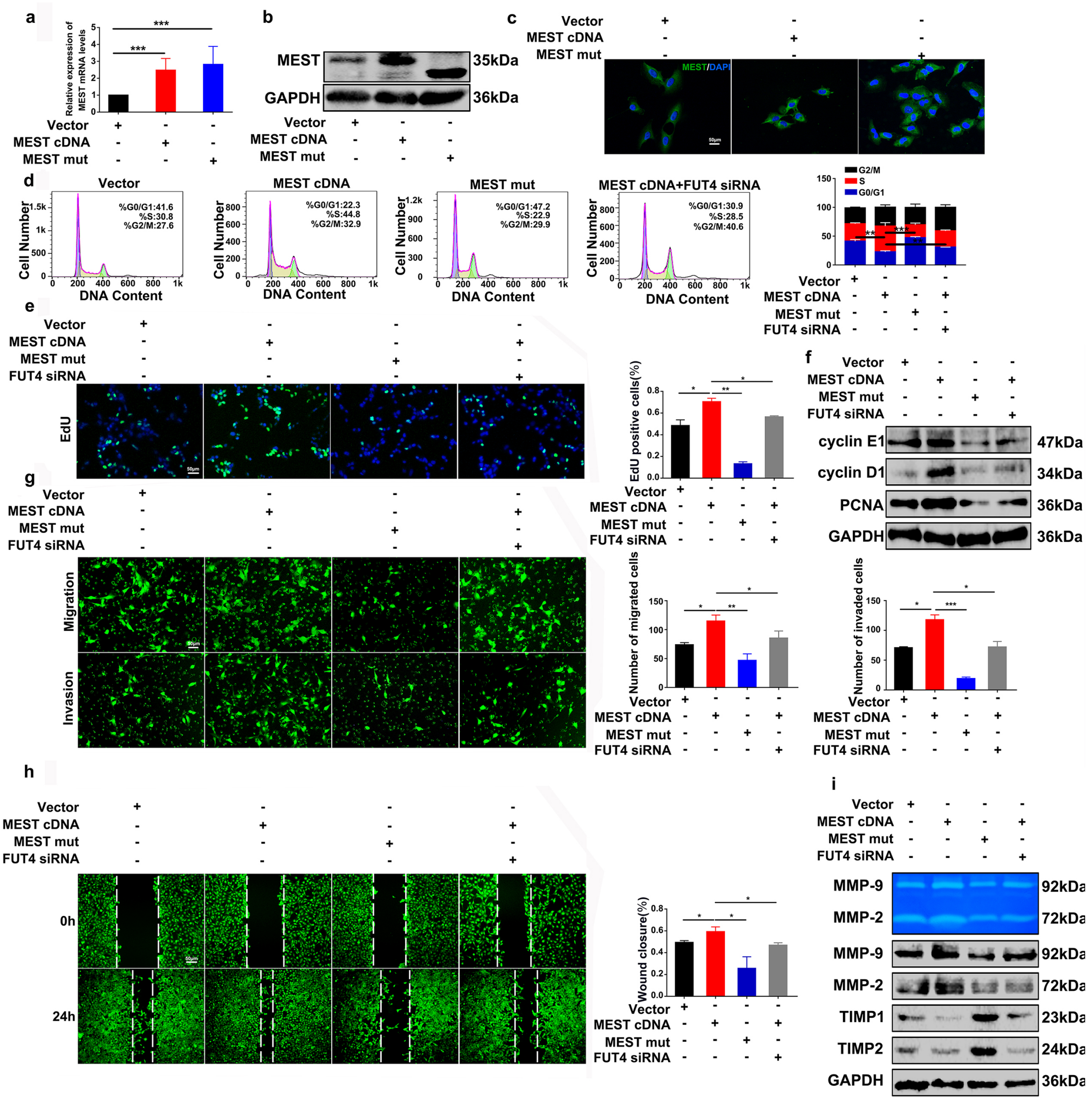

Supplementary Figure 3. FUT4 siRNA inhibits LeY biosynthesis, further impairs trophoblast implantation potential. (a, b) Real-time PCR and western blot analysis of MEST at the mRNA level (a) and protein level (b). **(c)** Immunofluorescence staining of MEST on HTR-8/SVneo cells after transfected with vector, MEST cDNA or MEST mut plasmid. **(d)** The cell cycle arrest phases were assessed by Flow cytometry in HTR-8/SVneo cells after transfected with vector, MEST cDNA, MEST mut plasmid or cotransfected MEST cDNA and FUT4 siRNA. **(e)** Edu assay evaluated cell proliferation potential. **(f)** Western blot detected the change of cyclin E1, D1 and PCNA. **(g, h)** Matrigel transwell

assay and Cell scratch test detected cell invading ability of HTR-8/SVneo cells. **(i)** Gelatin zymography assay and western blot detected the activation and alteration of MMPs and TIMP-1/2. *$p < 0.05$, **$p < 0.01$, ***$p < 0.001$

Supplementary Figure 4

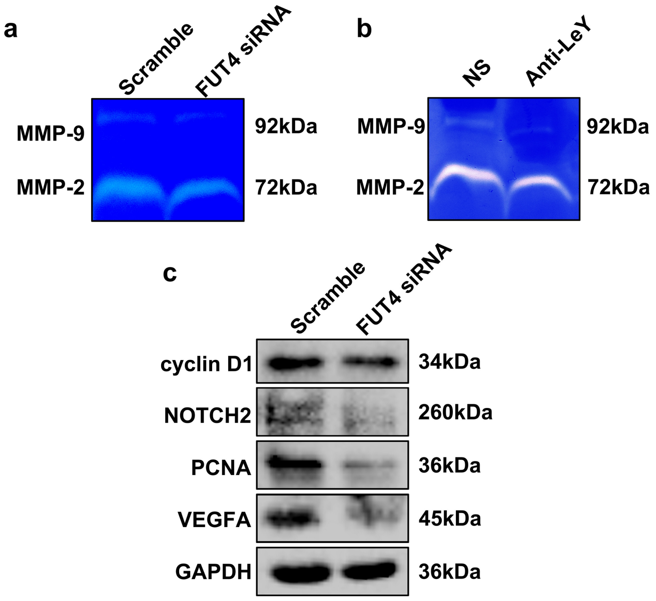

Supplementary Figure 4. Silencing FUT4 and anti LeY antibody blockage inhibit MMPs secretion and implantation relation protein expression of embryo *in vivo*. Mice embryo transfected with scramble RNA, FUT4 siRNA, or treated with normal saline (NS) or anti-LeY antibody, respectively, culture medium and outgrowth trophoblast were collected for detection. **(a, b)** Gelatin zymography assay detected the activation MMP2/9 of cultured medium. **(c)** Western blot detected the change of cyclin D1, NOTCH2, PCNA and VEGFA in outgrowth trophoblast.

Supplementary Table. 1

Symbol	GO Term
UniProtKB - Q5EB52~MEST	GO:0003824 catalytic activity
	GO:0005783 endoplasmic reticulum
	GO:0016020 membrane
	GO:0016021 integral component of membrane
	GO:0016787 hydrolase activity
	GO:0007498 mesoderm development
	GO:0005515 protein binding
	GO:0005789 endoplasmic reticulum membrane
	GO:0070062 extracellular exosome
	GO:0005783 endoplasmic reticulum
	GO:0010883 regulation of lipid storage
	GO:0005783 endoplasmic reticulum
UniProtKB - P0DML2~CSH1	GO:0005179 hormone activity
	GO:0005576 extracellular region
	GO:0046872 metal ion binding
	GO:0005783 endoplasmic reticulum
	GO:0031982 vesicle
	GO:0005576 extracellular region
	GO:0031904 endosome lumen
	GO:0005515 protein binding
	GO:0008083 growth factor activity
	GO:0031667 response to nutrient levels
	GO:0046427 positive regulation of receptor signaling pathway via JAK-STAT
	GO:0045927 positive regulation of growth
	GO:0042531 positive regulation of tyrosine phosphorylation of STAT protein
	GO:0005179 hormone activity
UniProtKB - P02647~APOA1	GO:0034364 high-density lipoprotein particle C
	GO:0005102 signaling receptor binding F

	GO:0001540 amyloid-beta binding F
	GO:0015485 cholesterol binding F
	GO:0030300 regulation of intestinal cholesterol absorption P
	GO:0005543 phospholipid binding F
	GO:0034380 high-density lipoprotein particle assembly P
	GO:0034372 very-low-density lipoprotein particle remodeling P
	GO:0033700 phospholipid efflux P
	GO:0033344 cholesterol efflux P
	GO:0010898 positive regulation of triglyceride catabolic process P
	GO:0010873 positive regulation of cholesterol esterification P
	GO:0006695 cholesterol biosynthetic process P
	GO:0031210 phosphatidylcholine binding F
	GO:0042627 chylomicron C
	GO:0051006 positive regulation of lipoprotein lipase activity P
	GO:0046889 positive regulation of lipid biosynthetic process P
	GO:0046470 phosphatidylcholine metabolic process P
	GO:0045723 positive regulation of fatty acid biosynthetic process P
	GO:0060228 phosphatidylcholine-sterol O-acyltransferase activator activity F
	GO:0043691 reverse cholesterol transport P
	GO:0070653 high-density lipoprotein particle receptor binding F
	GO:0042632 cholesterol homeostasis P
	GO:0042157 lipoprotein metabolic process P
	GO:0070328 triglyceride homeostasis P

	GO:0005515 protein binding F
	GO:0018206 peptidyl-methionine modification P
	GO:0018158 protein oxidation P
	GO:0005576 extracellular region C
	GO:0006869 lipid transport P
	GO:0008289 lipid binding F
	GO:0008203 cholesterol metabolic process P
	GO:0008202 steroid metabolic process P
	GO:0006629 lipid metabolic process P
	GO:0030301 cholesterol transport P
	GO:0005615 extracellular space C
	GO:0051180 vitamin transport P
	GO:0005788 endoplasmic reticulum lumen C
	GO:0005769 early endosome C
	GO:0034774 secretory granule lumen C
	GO:0005829 cytosol C
	GO:0071682 endocytic vesicle lumen C
	GO:0005886 plasma membrane C
	GO:0042802 identical protein binding F
	GO:0005319 lipid transporter activity F
	GO:0030325 adrenal gland development P
	GO:0008035 high-density lipoprotein particle binding F
	GO:0019915 lipid storage P
	GO:0001935 endothelial cell proliferation P
	GO:0001932 regulation of protein phosphorylation P
	GO:0008211 glucocorticoid metabolic process P
	GO:0006644 phospholipid metabolic process P
	GO:0120020 cholesterol transfer activity F
	GO:0051346 negative regulation of hydrolase activity P
	GO:0071813 lipoprotein particle binding F
	GO:0043534 blood vessel endothelial cell migration P
	GO:0042158 lipoprotein biosynthetic process P

	**GO:0019899 enzyme binding F**
	**GO:0034384 high-density lipoprotein particle clearance P**
	**GO:0034375 high-density lipoprotein particle remodeling P**
	**GO:0034115 negative regulation of heterotypic cell-cell adhesion P**
	**GO:0031410 cytoplasmic vesicle C**
	**GO:0032691 negative regulation of interleukin-1 beta production P**
	**GO:0032489 regulation of Cdc42 protein signal transduction P**
	**GO:0007186 G protein-coupled receptor signaling pathway P**
	**GO:0006656 phosphatidylcholine biosynthetic process P**
	**GO:0010903 negative regulation of very-low-density lipoprotein particle remodeling P**
	**GO:0010804 negative regulation of tumor necrosis factor-mediated signaling pathway P**
	**GO:0002719 negative regulation of cytokine production involved in immune response P**
	**GO:0030139 endocytic vesicle C**
	**GO:0062023 collagen-containing extracellular matrix C**
	**GO:0034361 very-low-density lipoprotein particle C**
	**GO:0034366 spherical high-density lipoprotein particle C**
	**GO:0034190 apolipoprotein receptor binding F**
	**GO:0034191 apolipoprotein A-I receptor binding F**
	**GO:0051345 positive regulation of hydrolase activity P**
	**GO:0050821 protein stabilization P**
	**GO:0050728 negative regulation of inflammatory response P**
	**GO:0060761 negative regulation of response to cytokine stimulus P**

	GO:0060354 negative regulation of cell adhesion molecule production P
	GO:0055091 phospholipid homeostasis P
	GO:0070508 cholesterol import P
	GO:0120009 intermembrane lipid transfer P
	GO:0031072 heat shock protein binding F
	GO:1903561 extracellular vesicle C
	GO:0070062 extracellular exosome C
	GO:0007229 integrin-mediated signaling pathway P
	GO:0007179 transforming growth factor beta receptor signaling pathway P
	GO:0010875 positive regulation of cholesterol efflux P
	GO:0072562 blood microparticle C
	GO:0051496 positive regulation of stress fiber assembly P
	GO:0050919 negative chemotaxis P
	GO:0050766 positive regulation of phagocytosis P
	GO:0070371 ERK1 and ERK2 cascade P
	GO:0045499 chemorepellent activity F
	GO:1902995 positive regulation of phospholipid efflux P
	GO:0035025 positive regulation of Rho protein signal transduction P
	GO:1900026 positive regulation of substrate adhesion-dependent cell spreading P
**UniProtKB - P11464~PSG1**	GO:0005576 extracellular region C
	GO:0007565 female pregnancy P
	GO:0005515 protein binding F
