# Supplementary material for: α1,3-fucosylation of MEST promotes invasion potential of cytotrophoblast cells by activating translation initiation
Source: Cell Death Dis. 2023 Oct 6;14(10):651. doi: 10.1038/s41419-023-06166-4 (PMC10556033; doi:10.1038/s41419-023-06166-4)
Supplement: Supplementary file 3 — Original Data File WB [file 41419_2023_6166_MOESM3_ESM.pdf]

Fig1 D

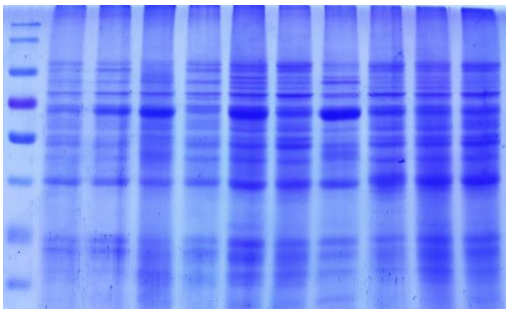

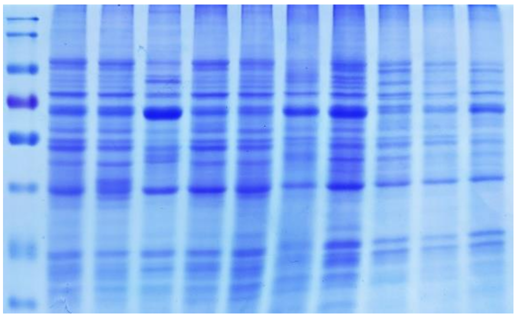

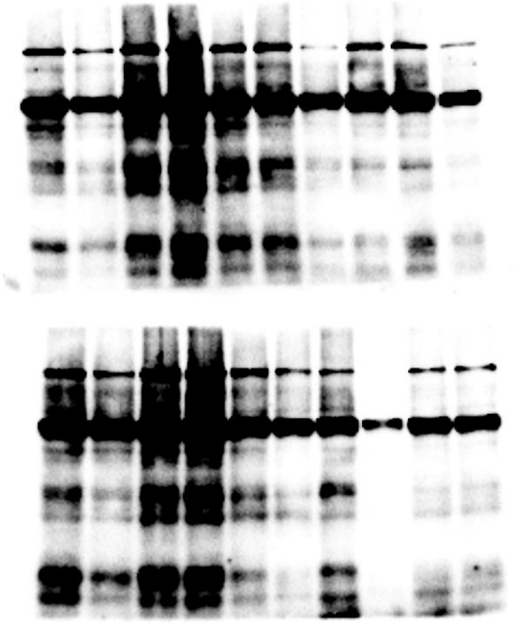

Fig1 G

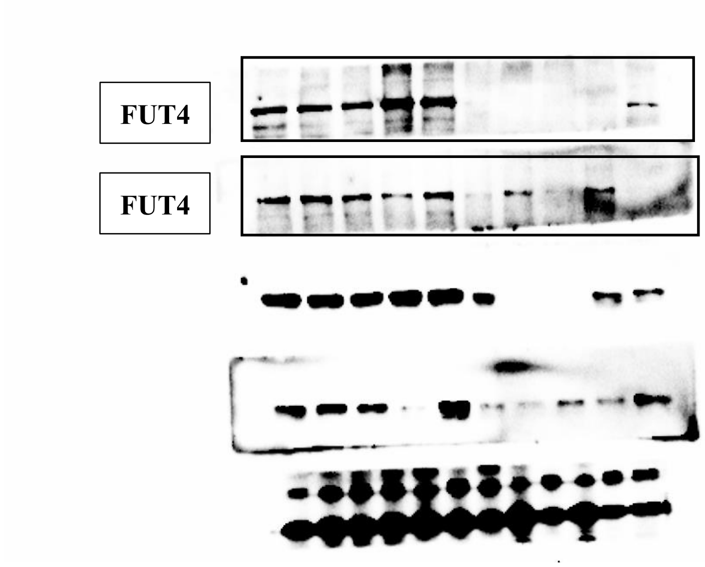

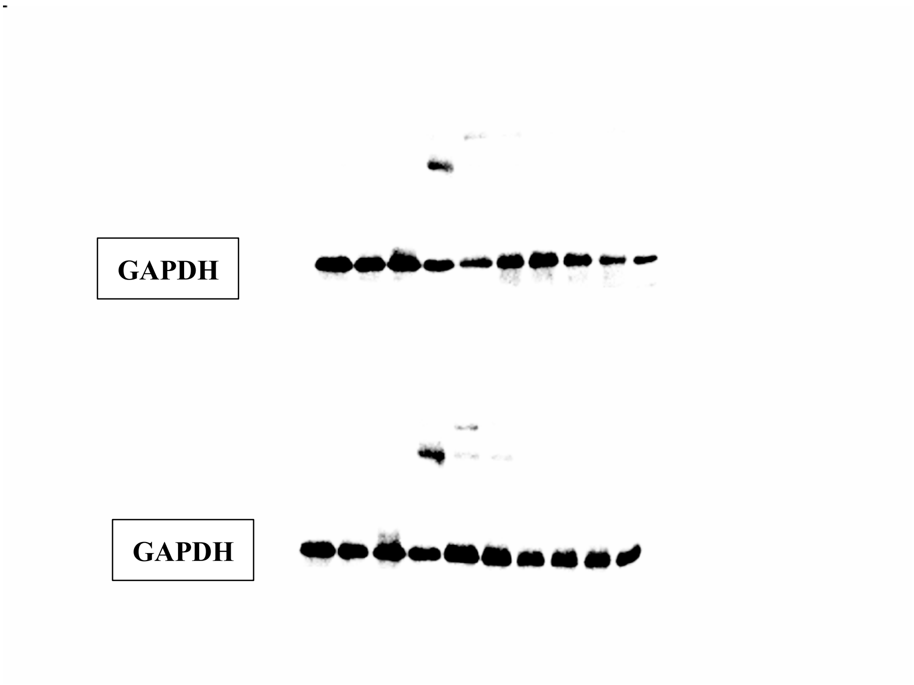

Fig1 I

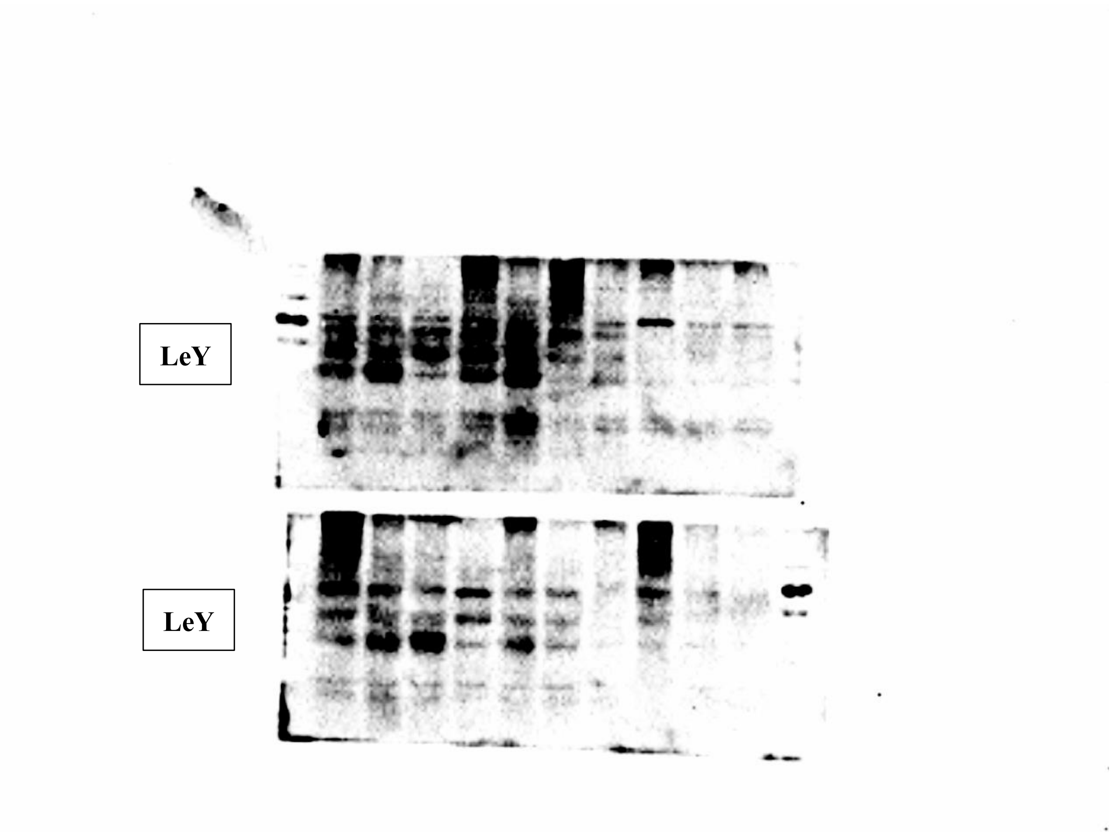

Fig2B

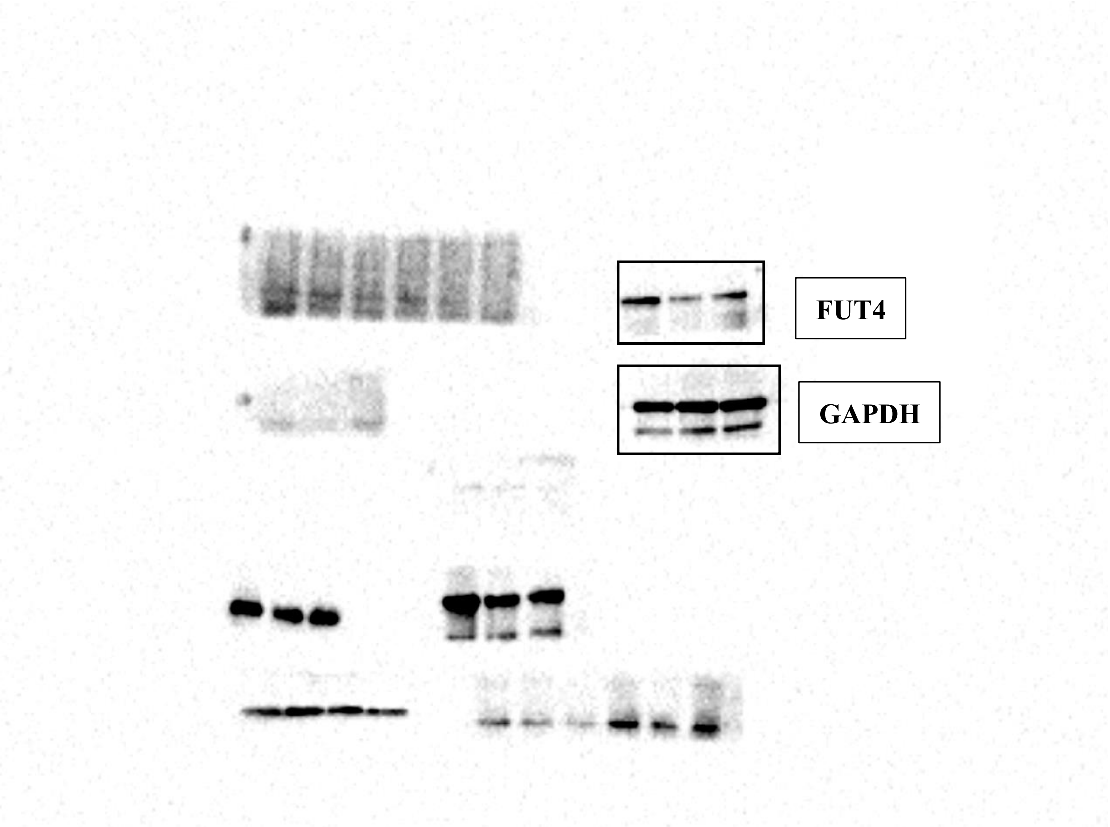

Fig2C

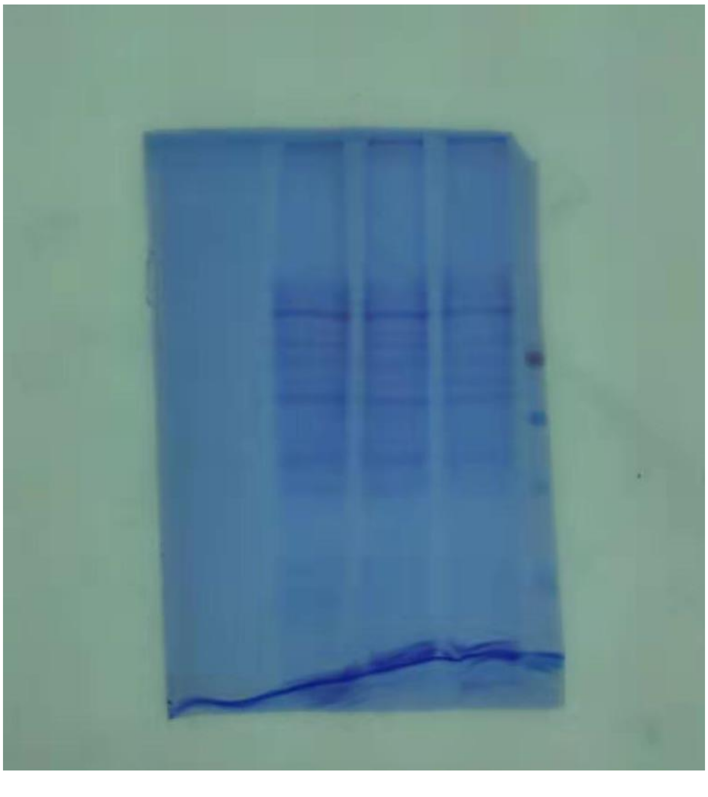

LeY

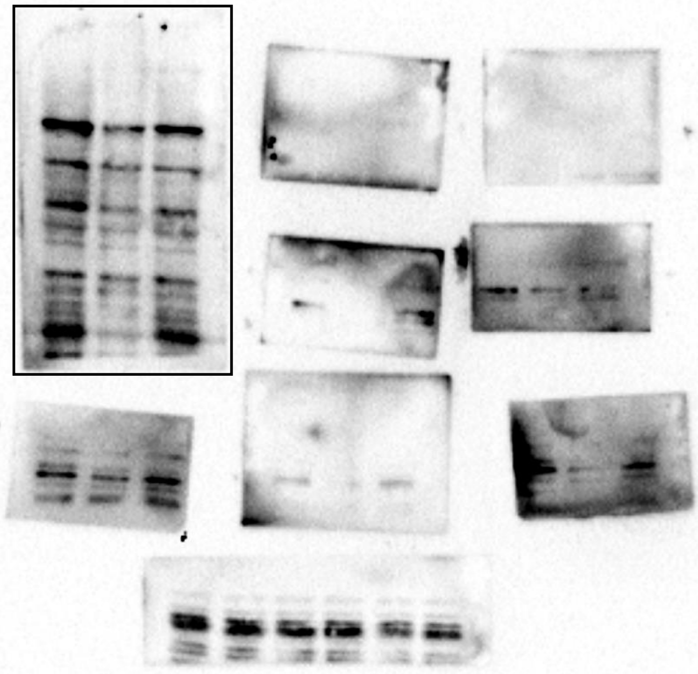

LTL

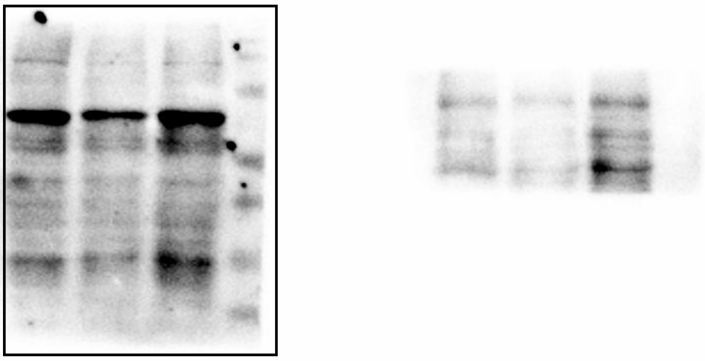

Fig2H

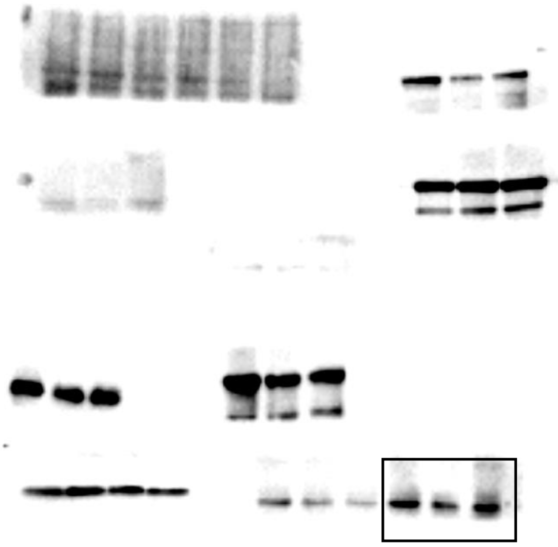

cyclin E1

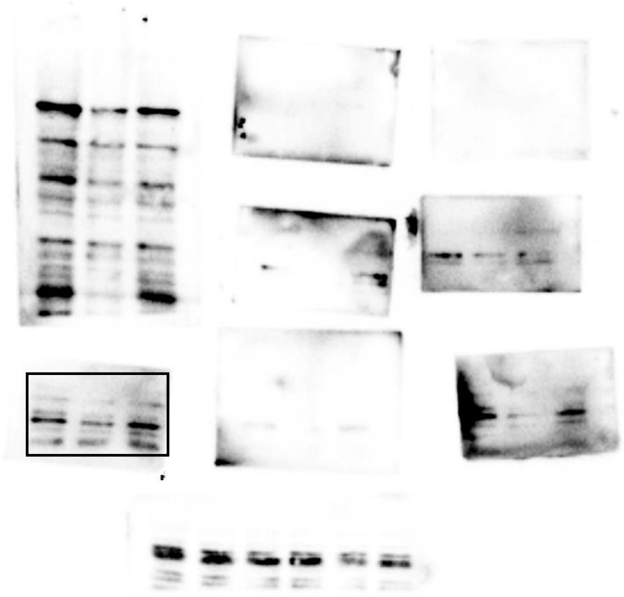

cyclin D1

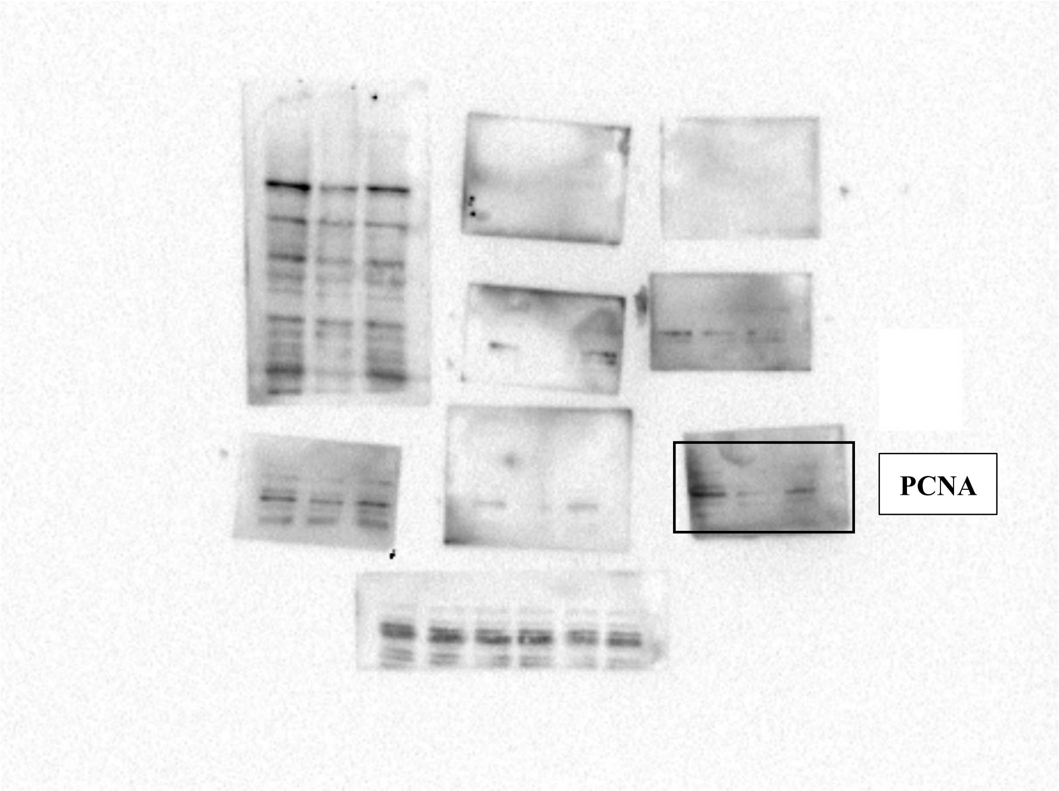
PCNA

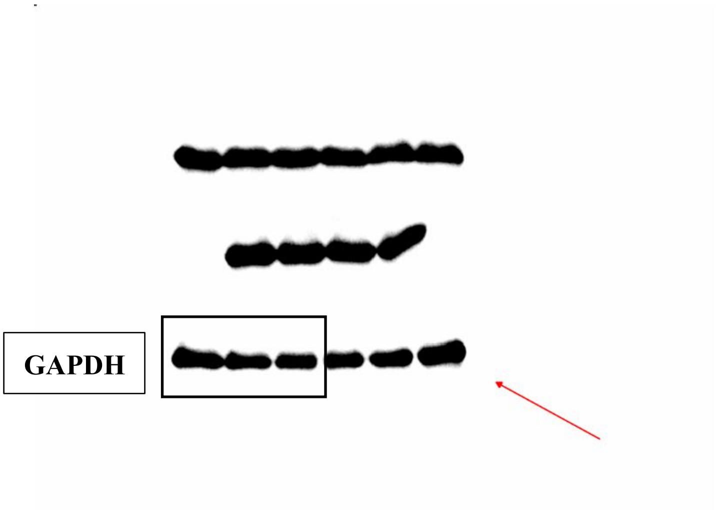
GAPDH

Fig2K

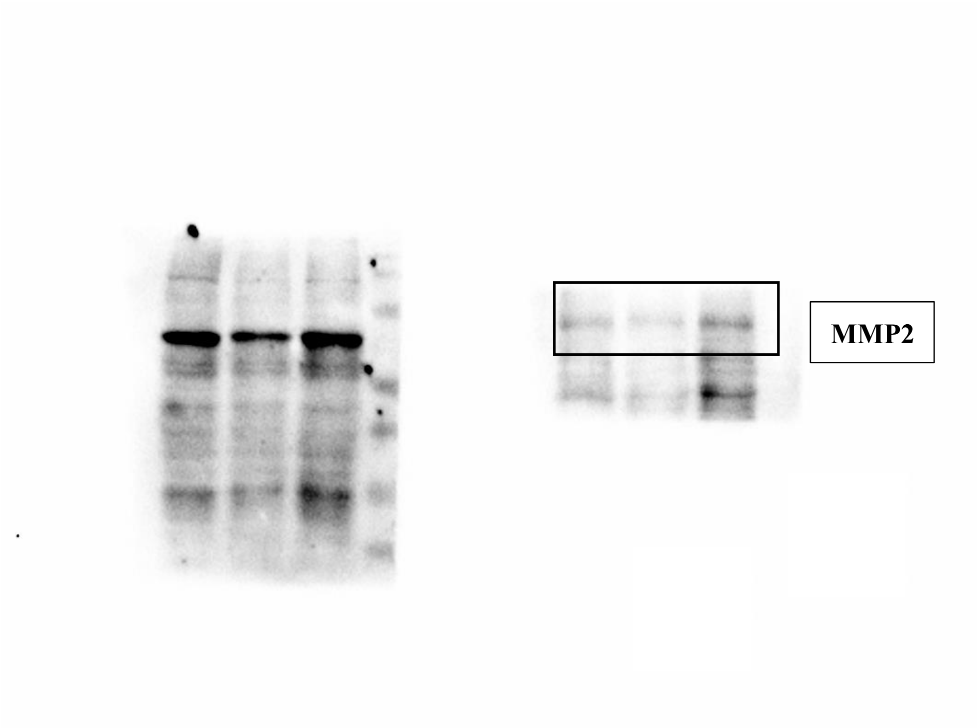

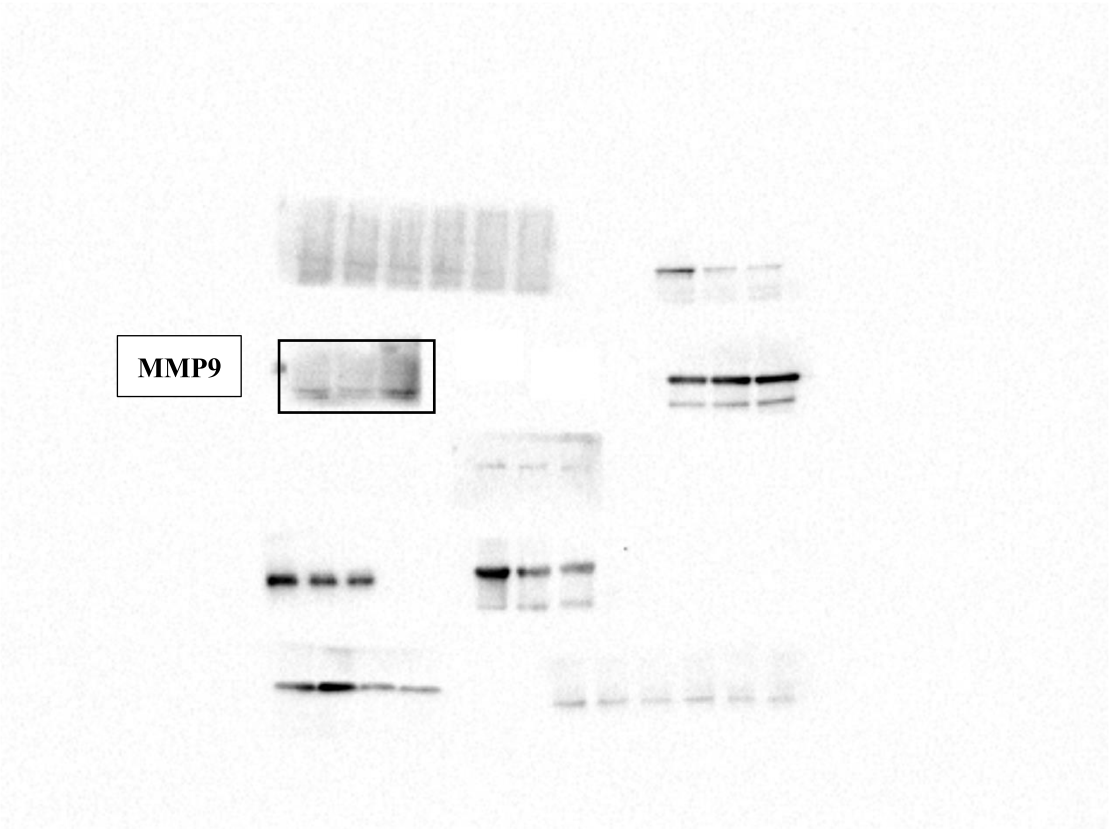

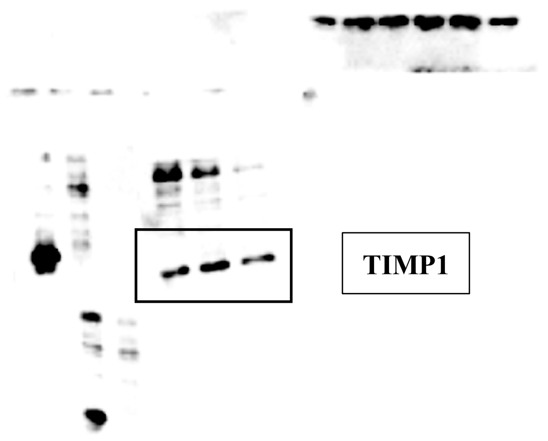
TIMP1

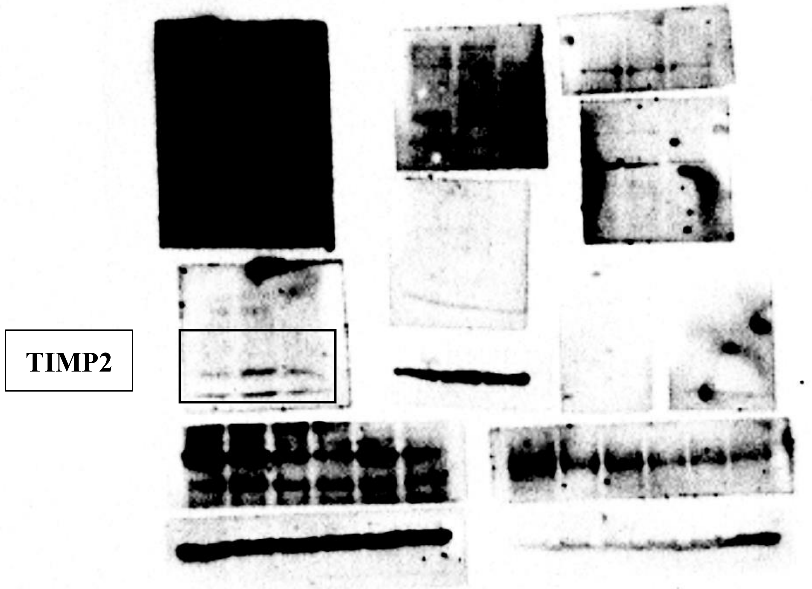
TIMP2

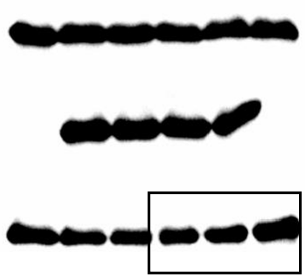

GAPDH

Fig3D

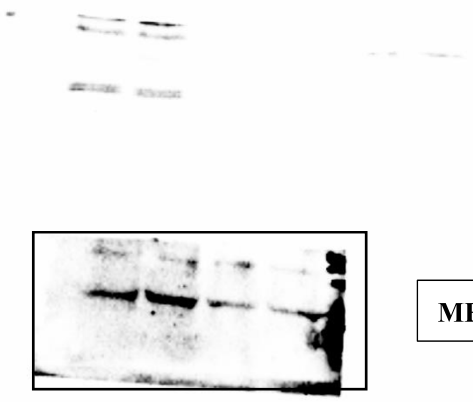

MEST

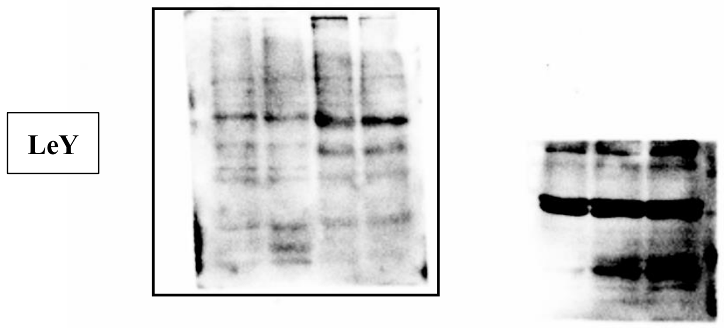

Fig3E

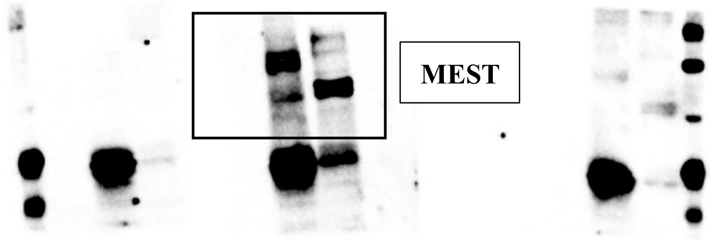

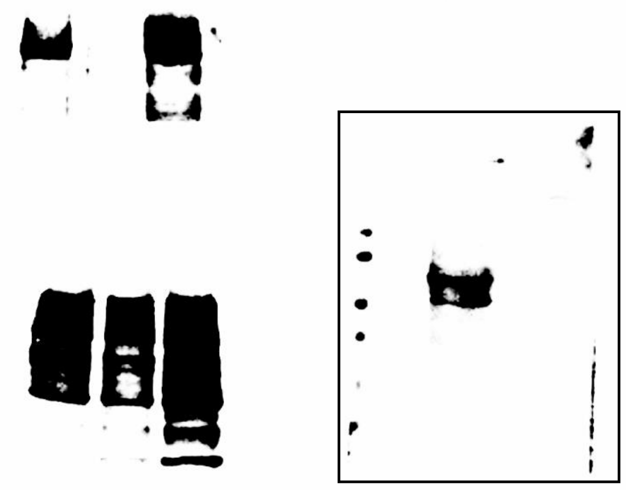

LeY

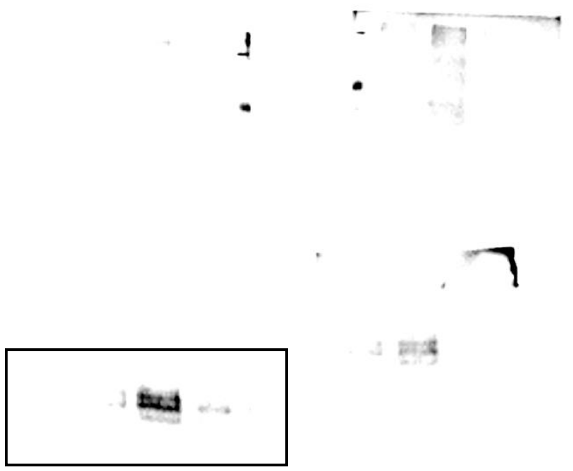

LTL

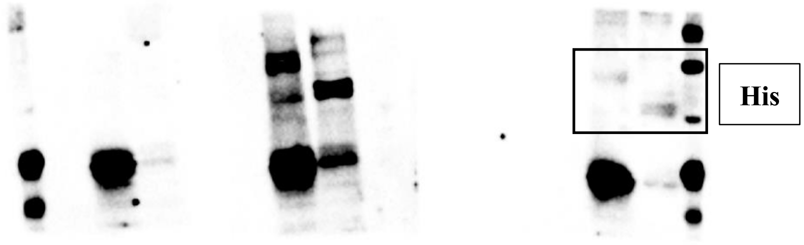

Fig3F

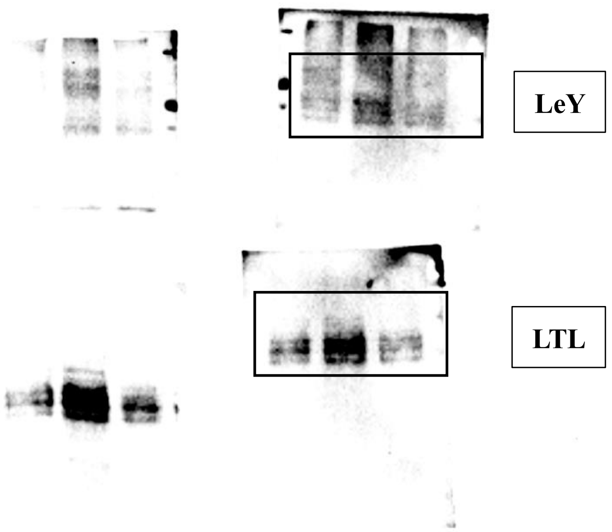

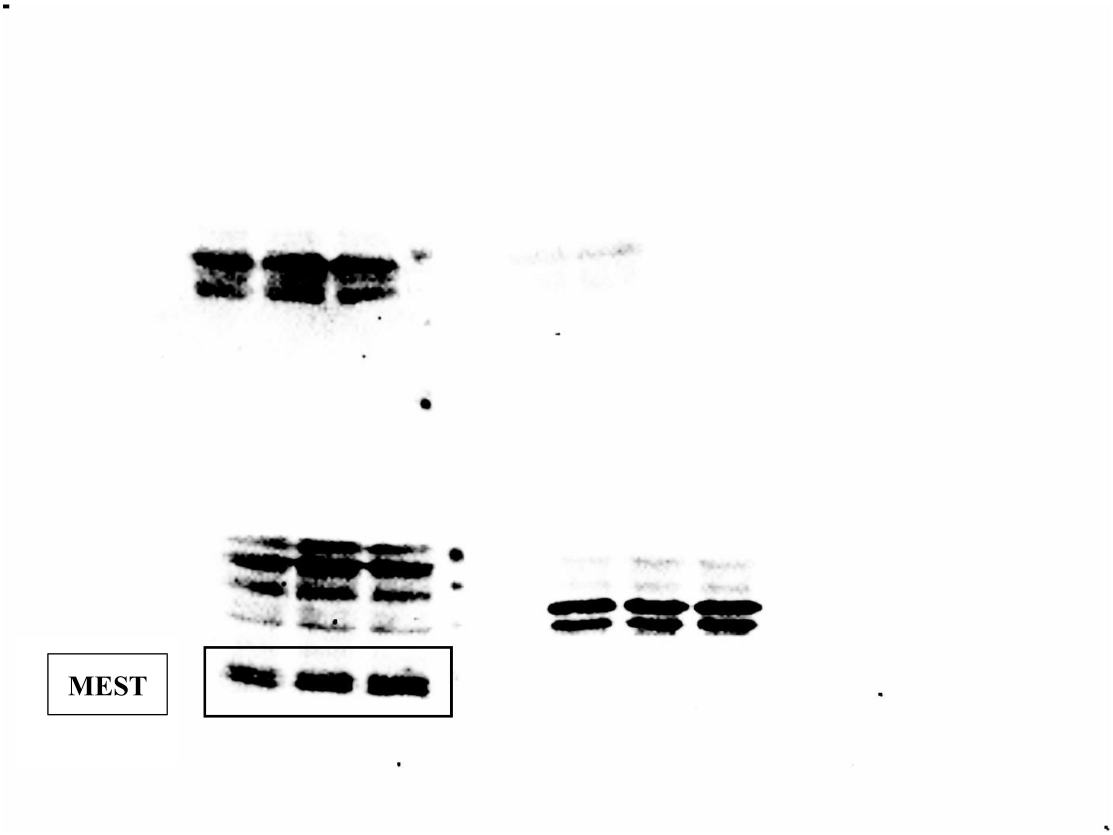

Fig4E

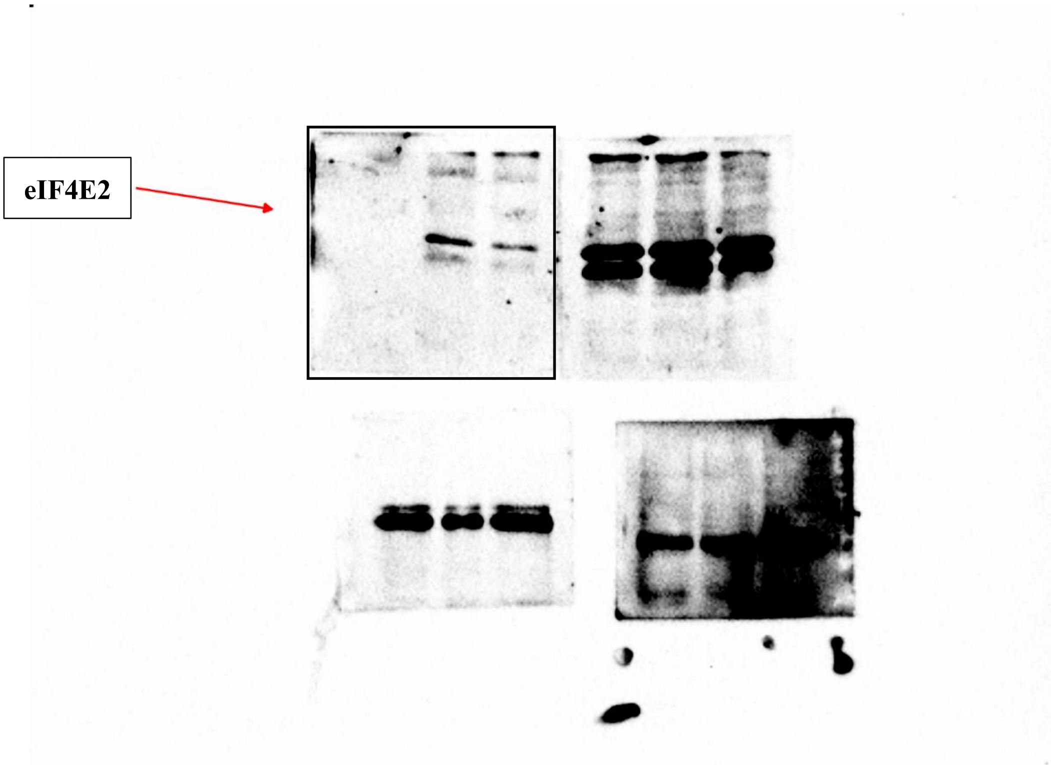

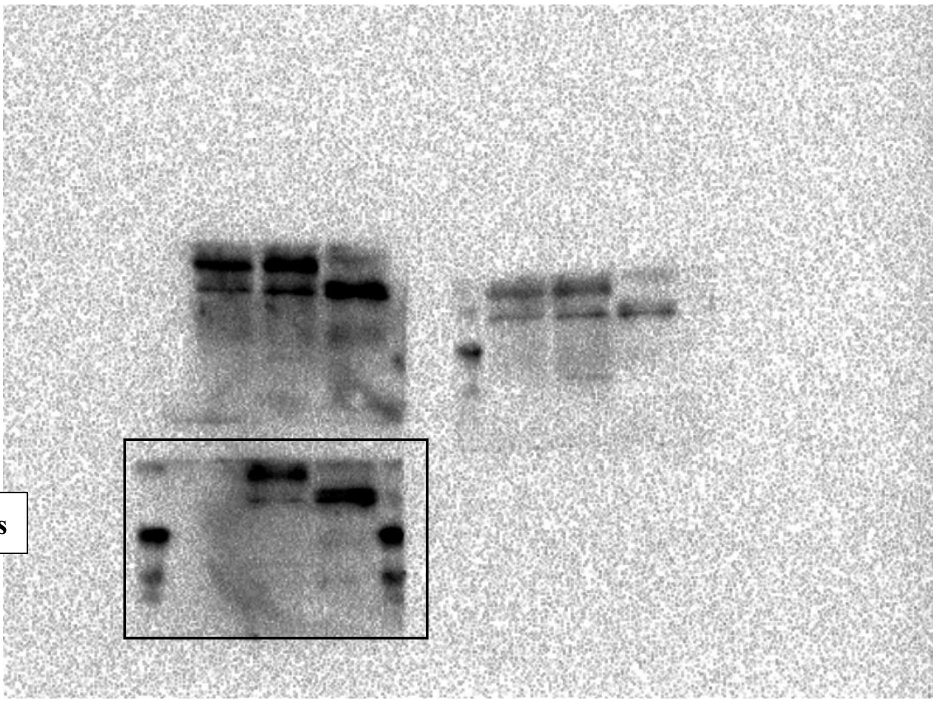

His

Fig4F

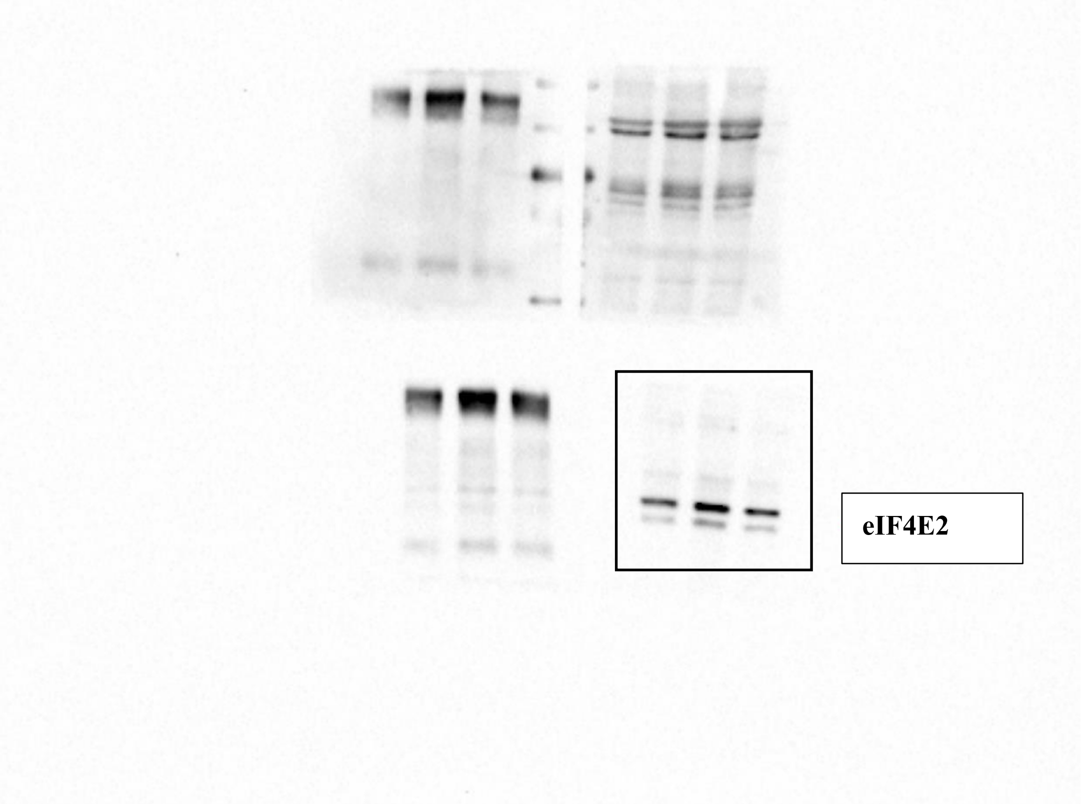

eIF4E2

Fig5F

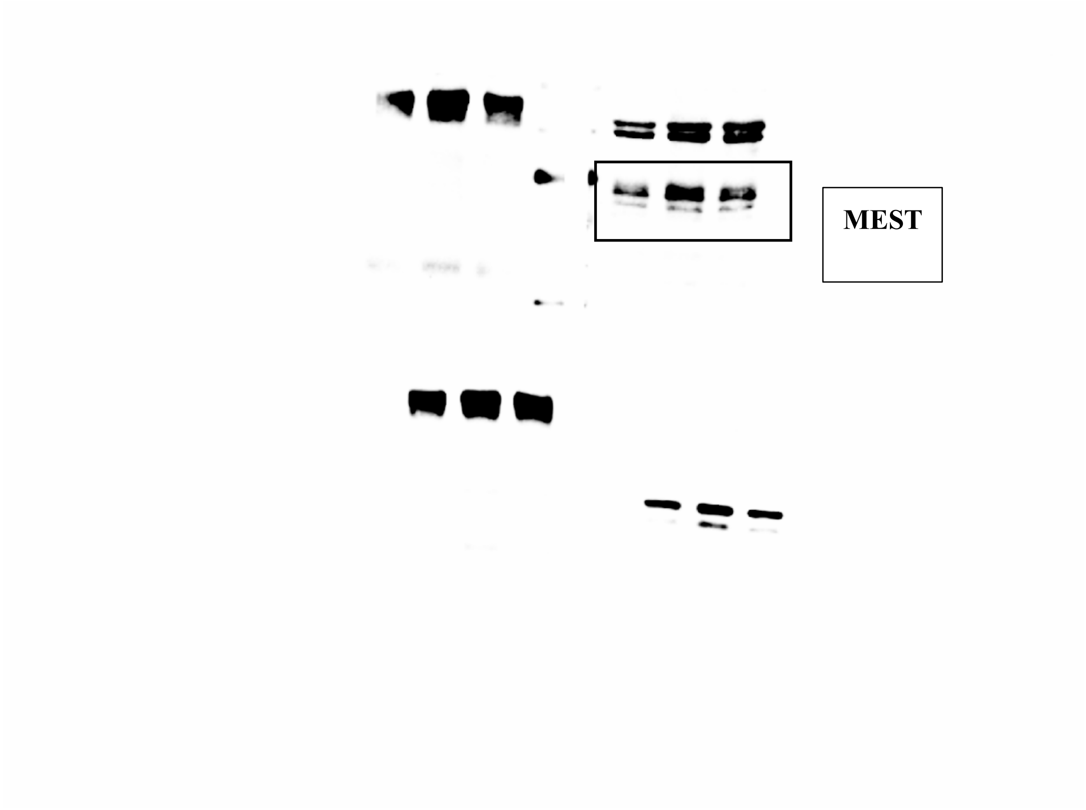

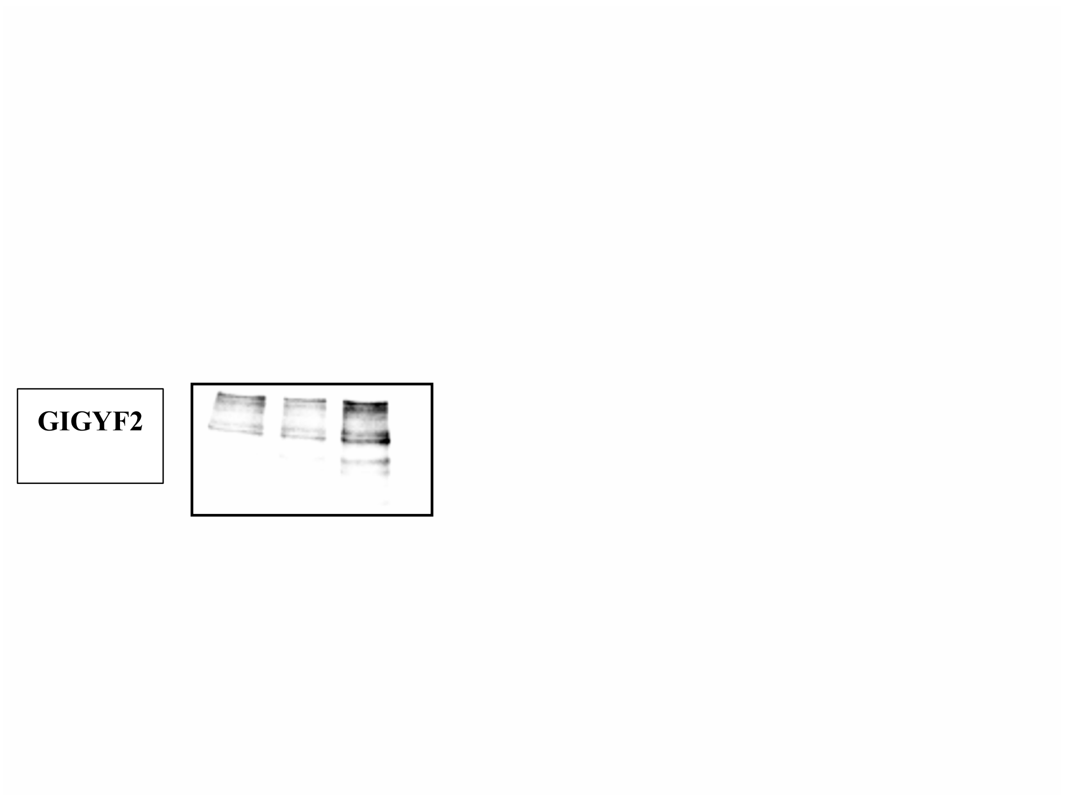

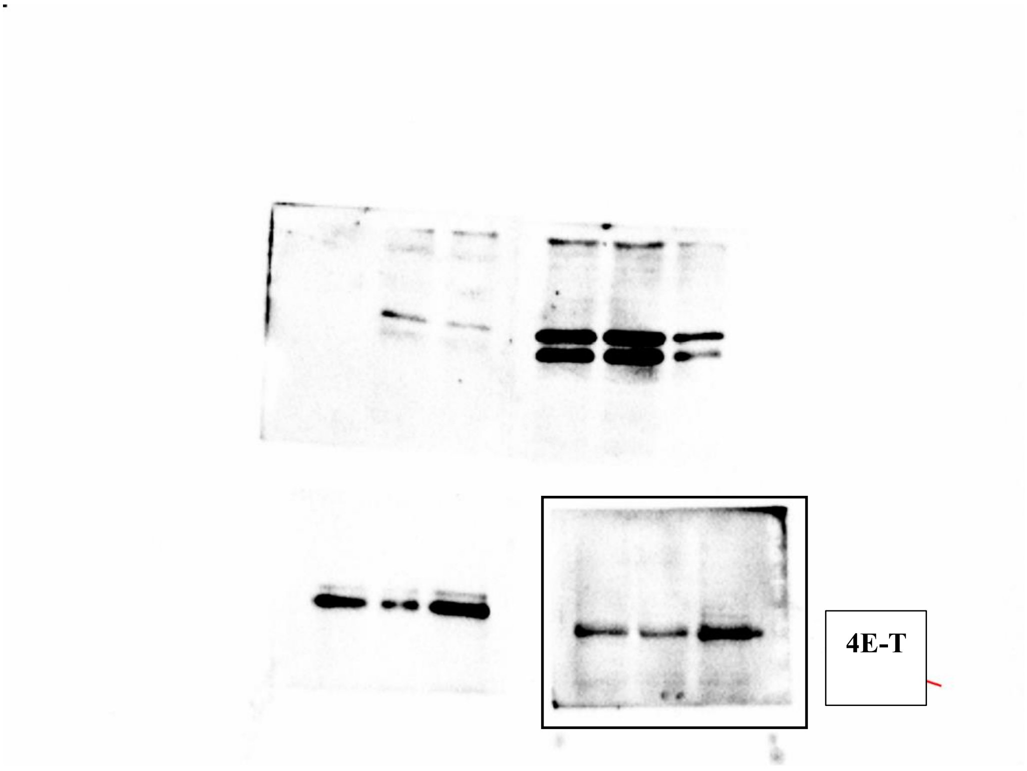
4E-T

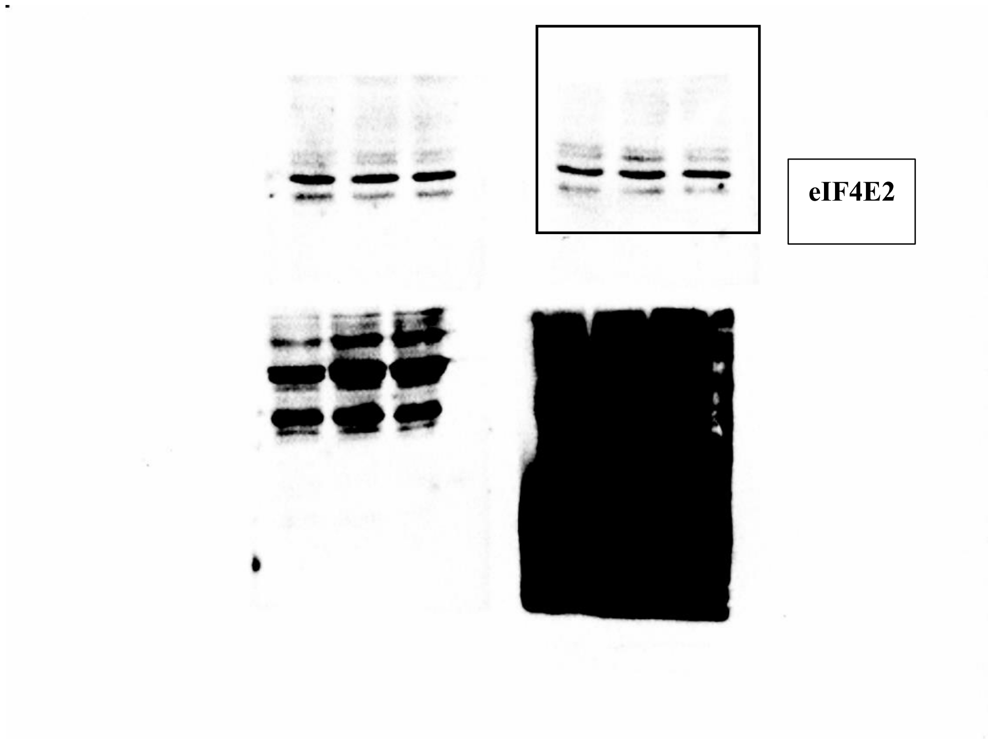
eIF4E2

Fig5G

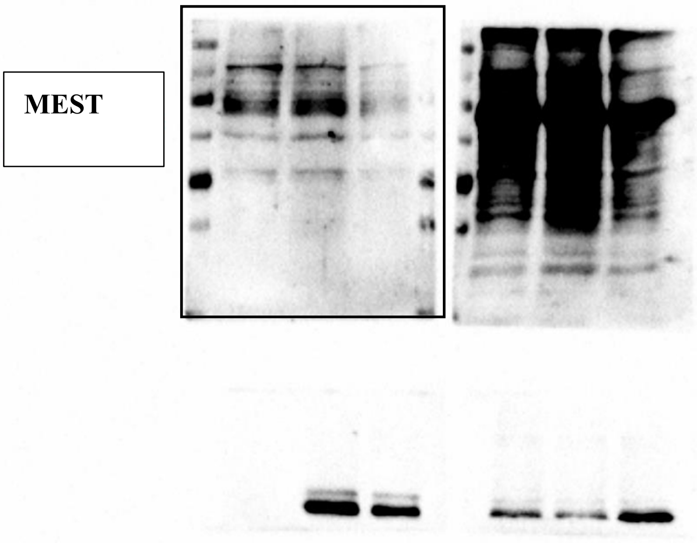

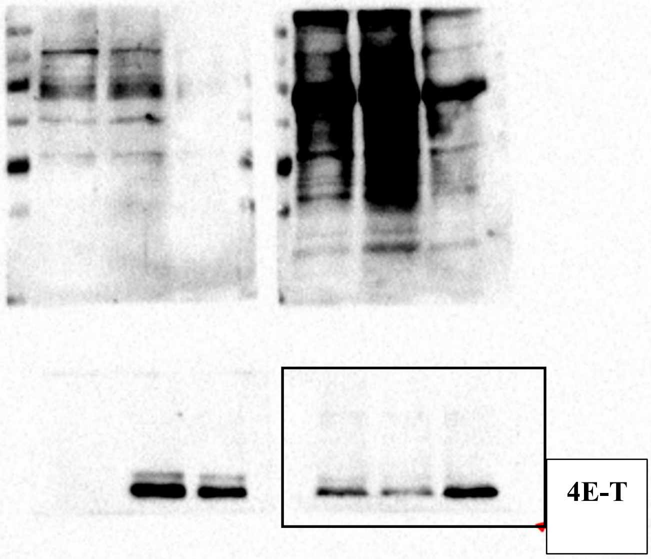

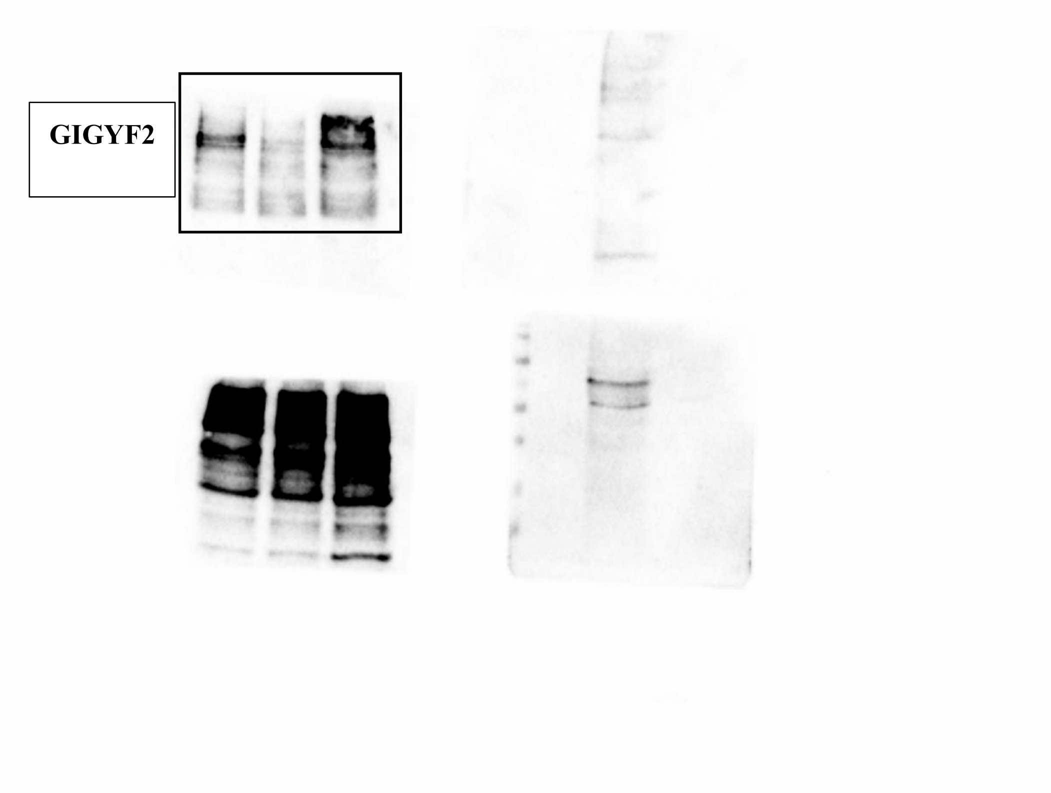
GIGYF2

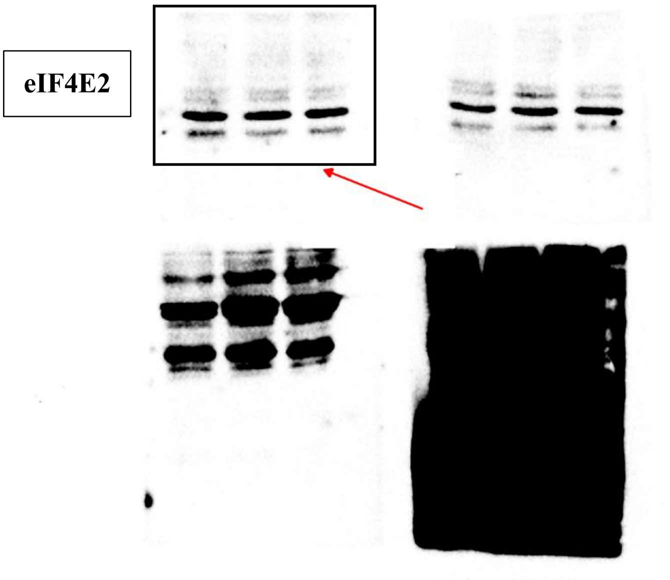
eIF4E2

Fig7C

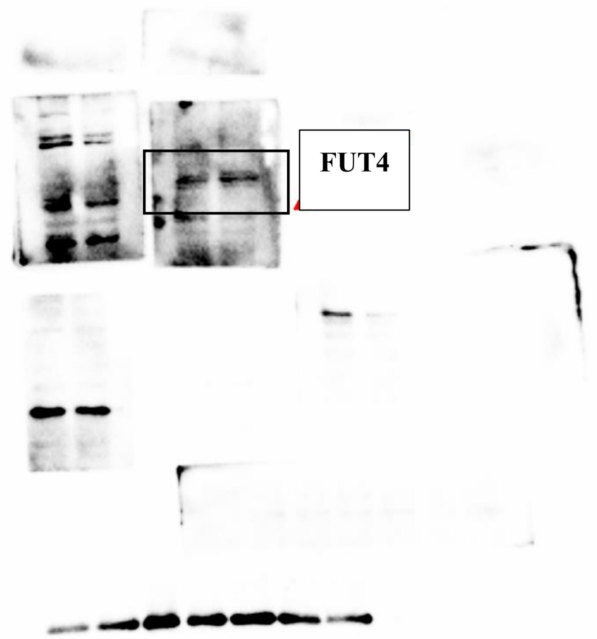

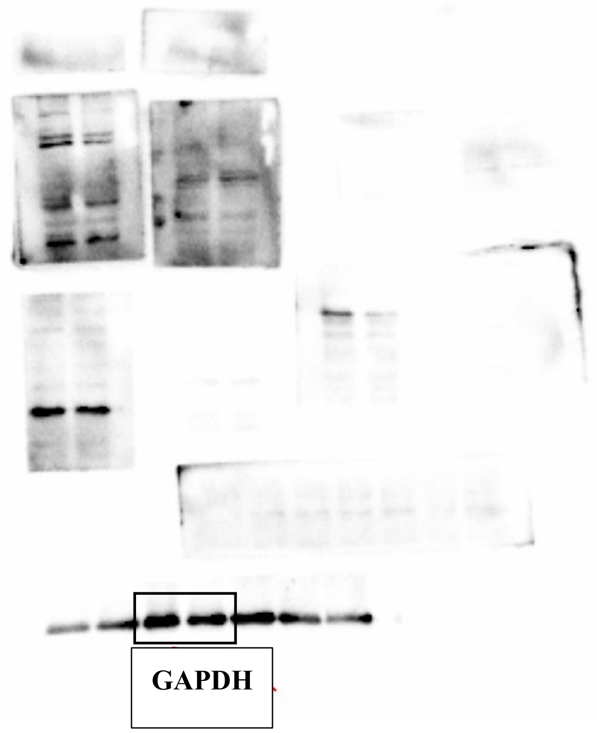

Fig7D

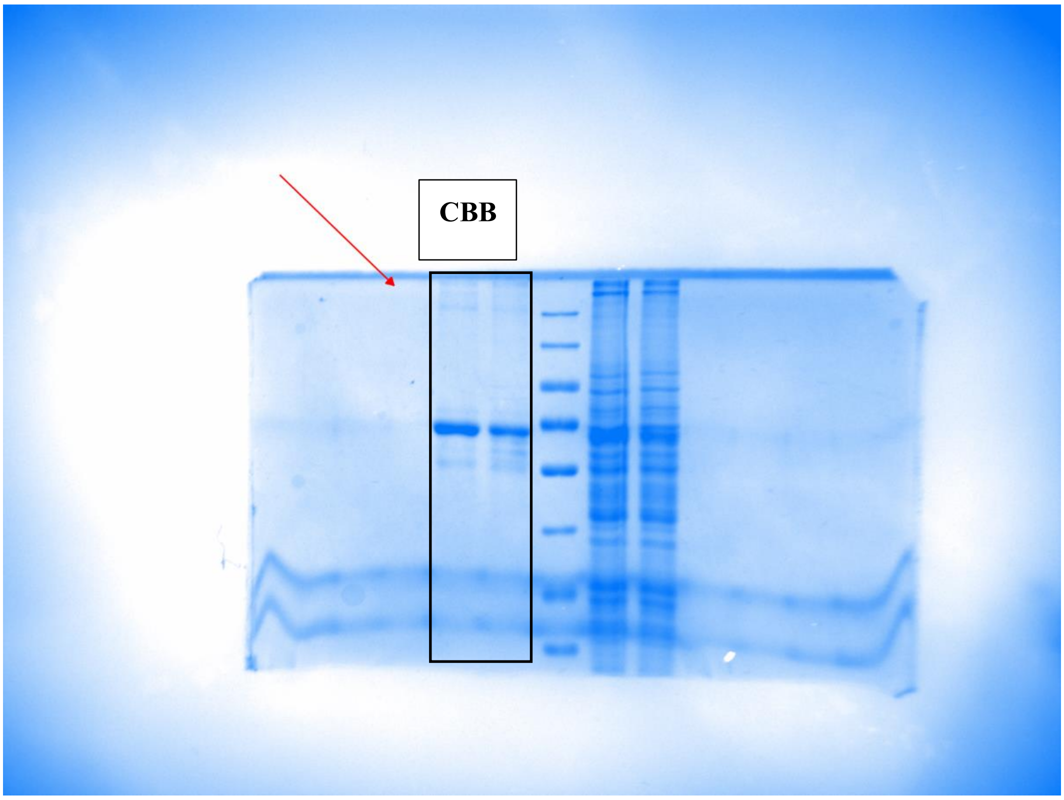

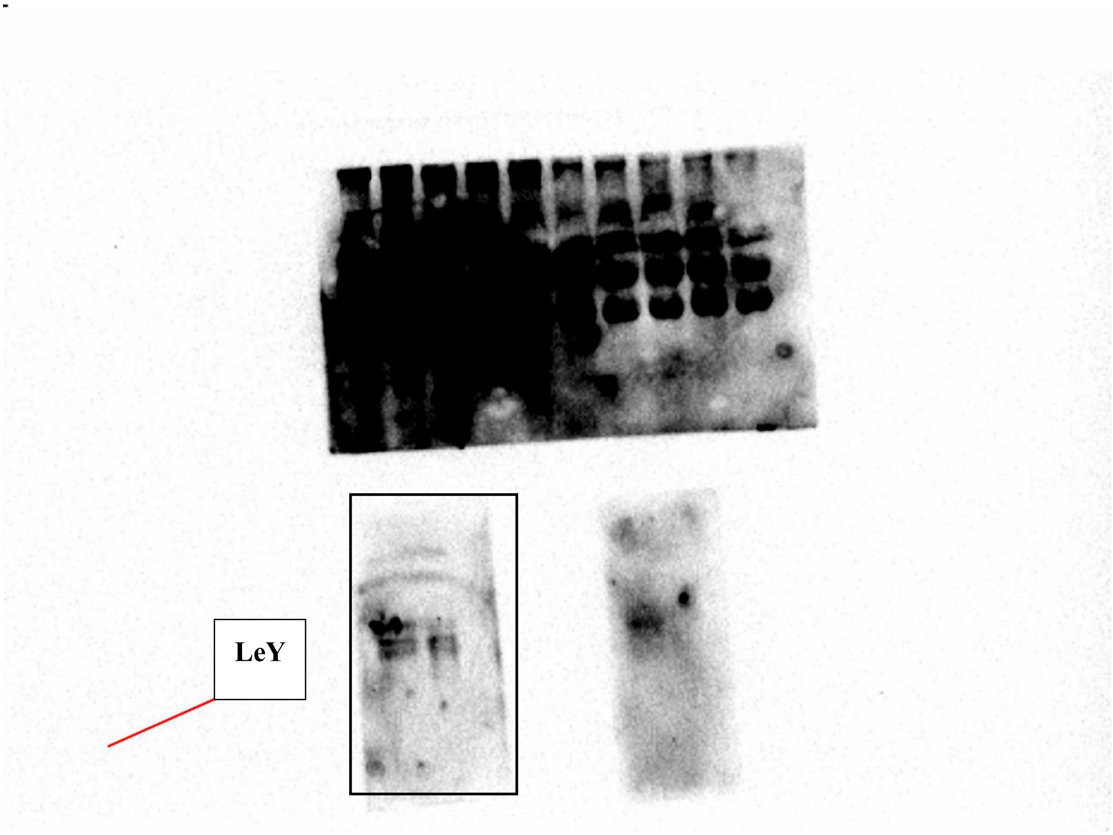

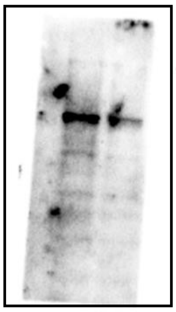

LTL

supplementary Fig.1A

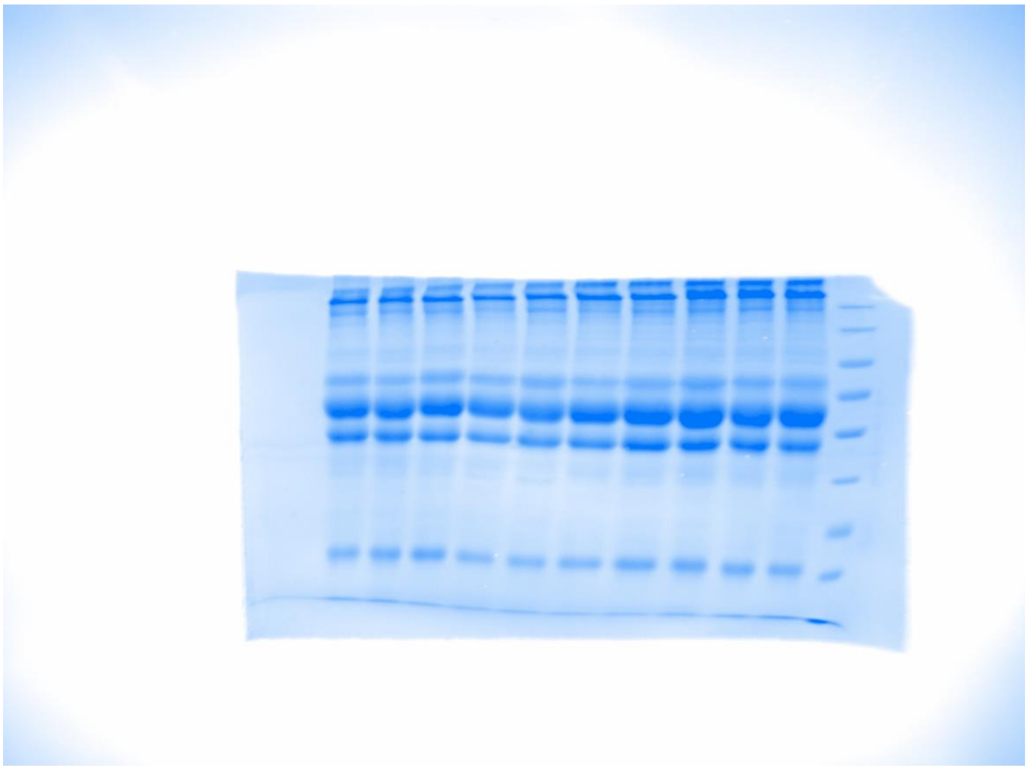

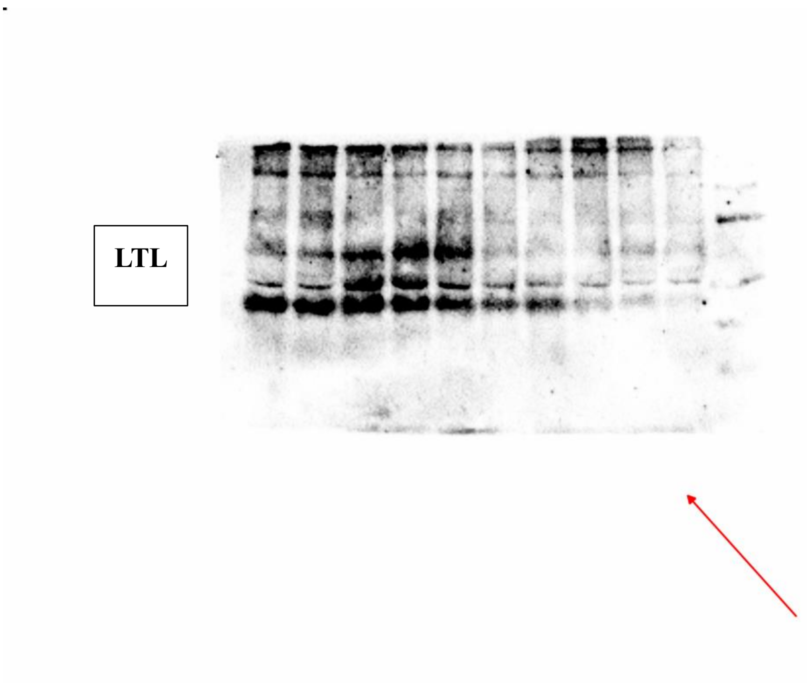
LTL

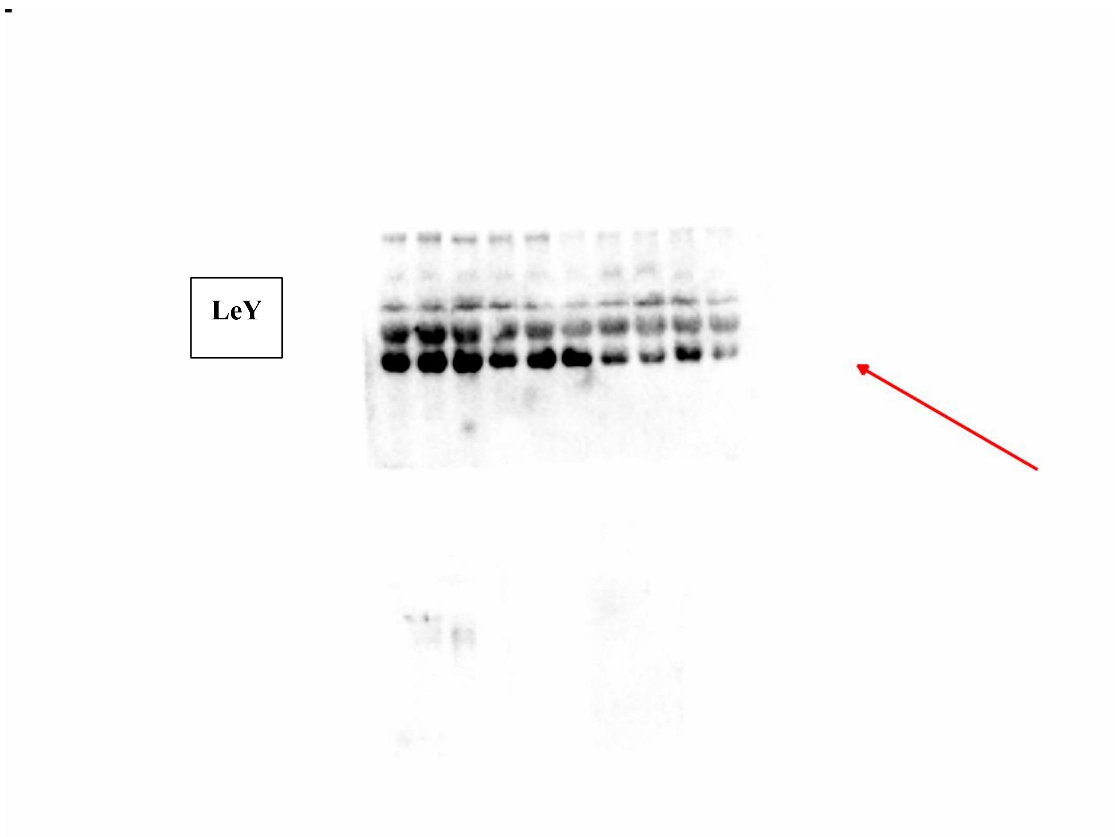
LeY

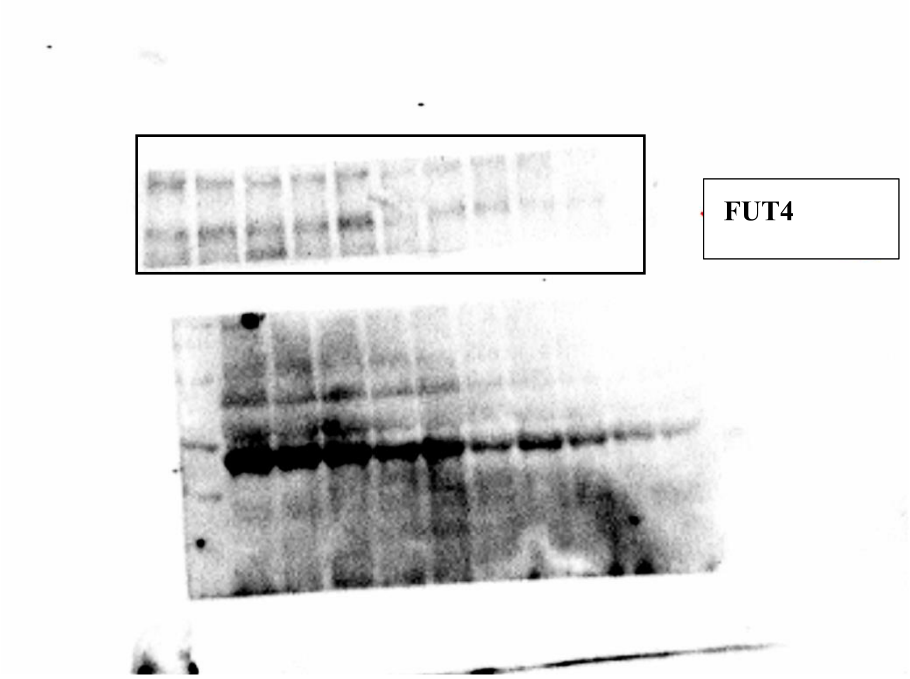

supplementary Fig.2B

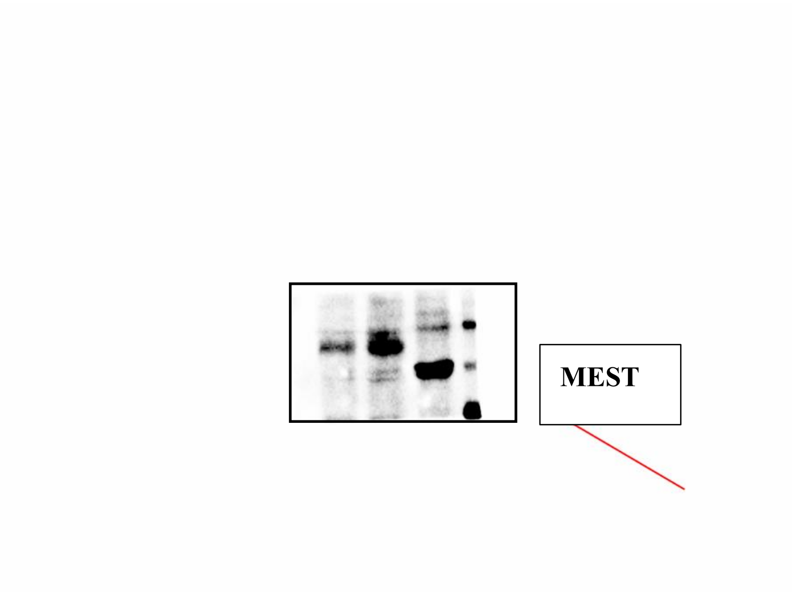

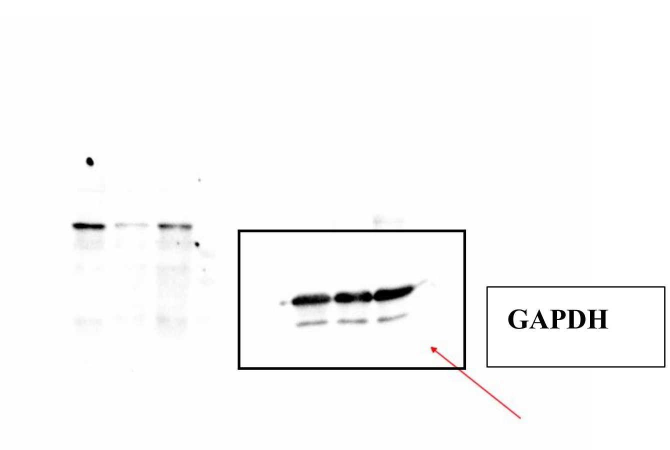

supplementary Fig.2F

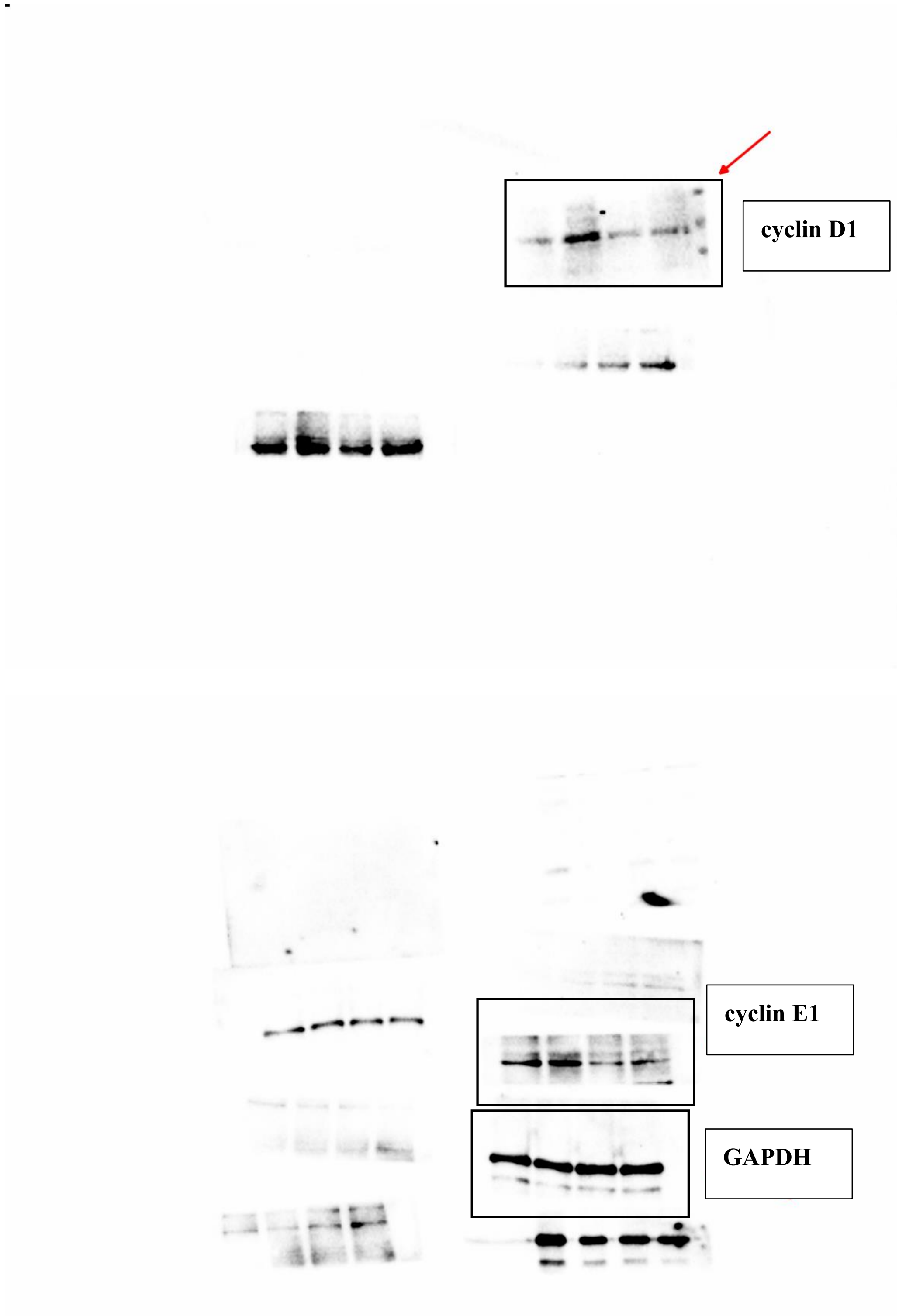

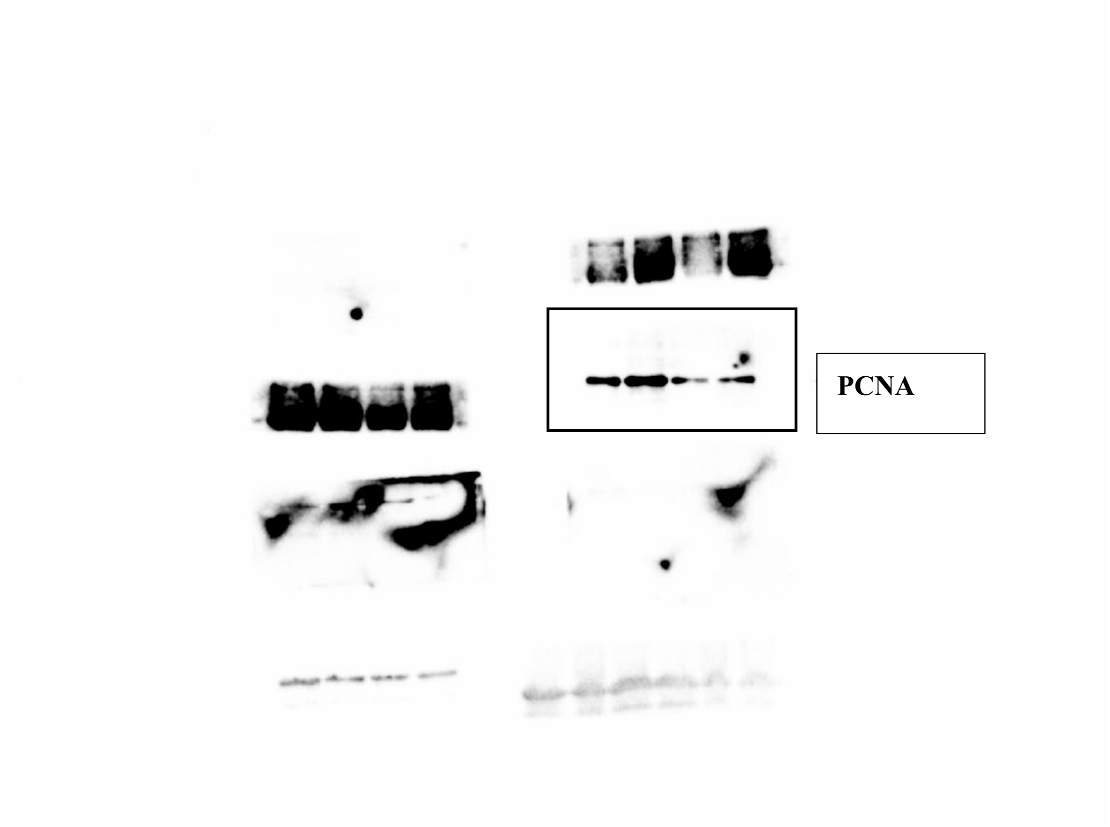

supplementary Fig.2I

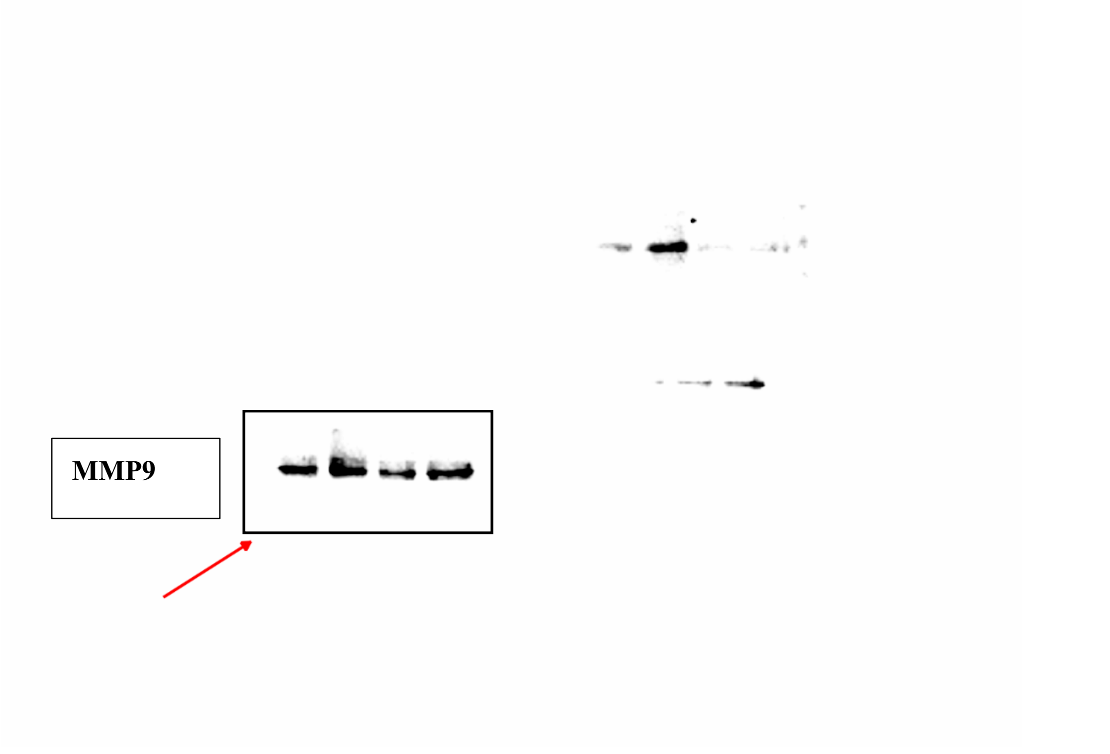

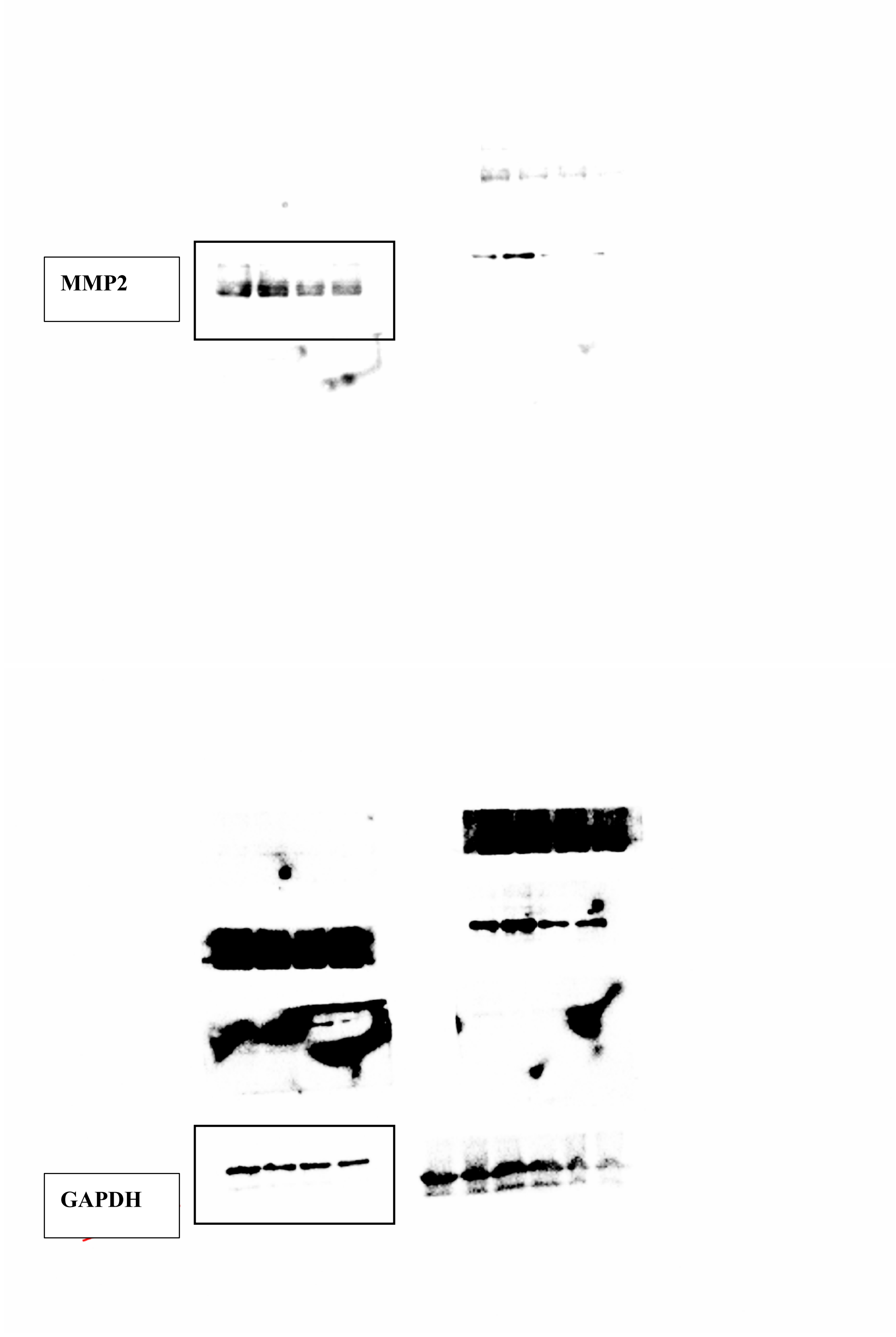
MMP2
GAPDH

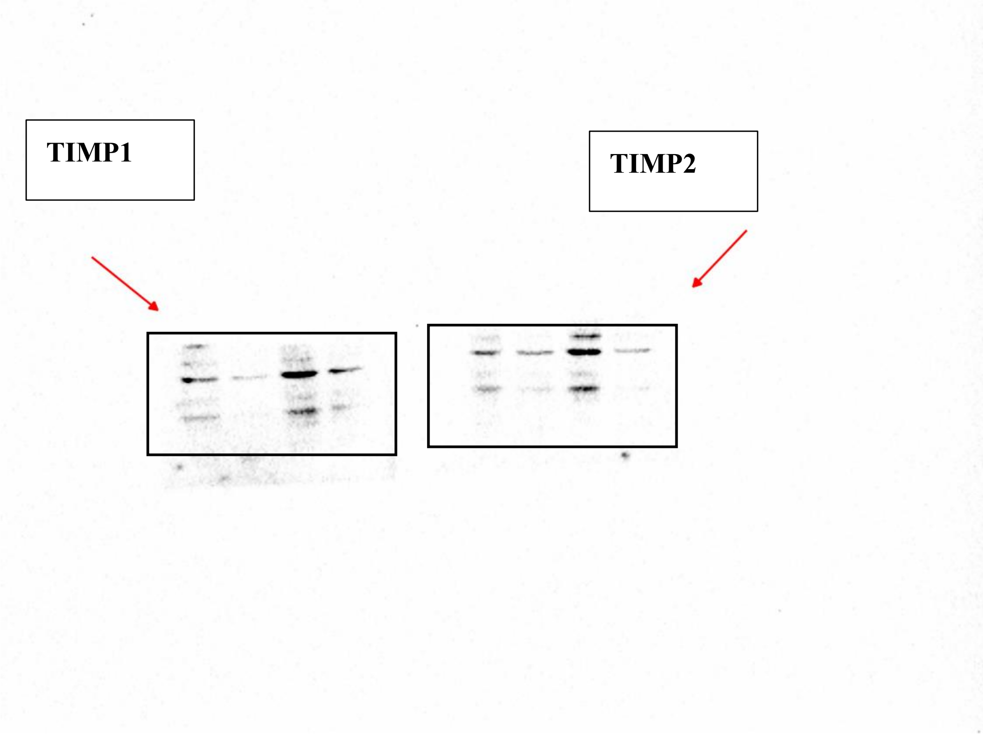

supplementary Fig.3C

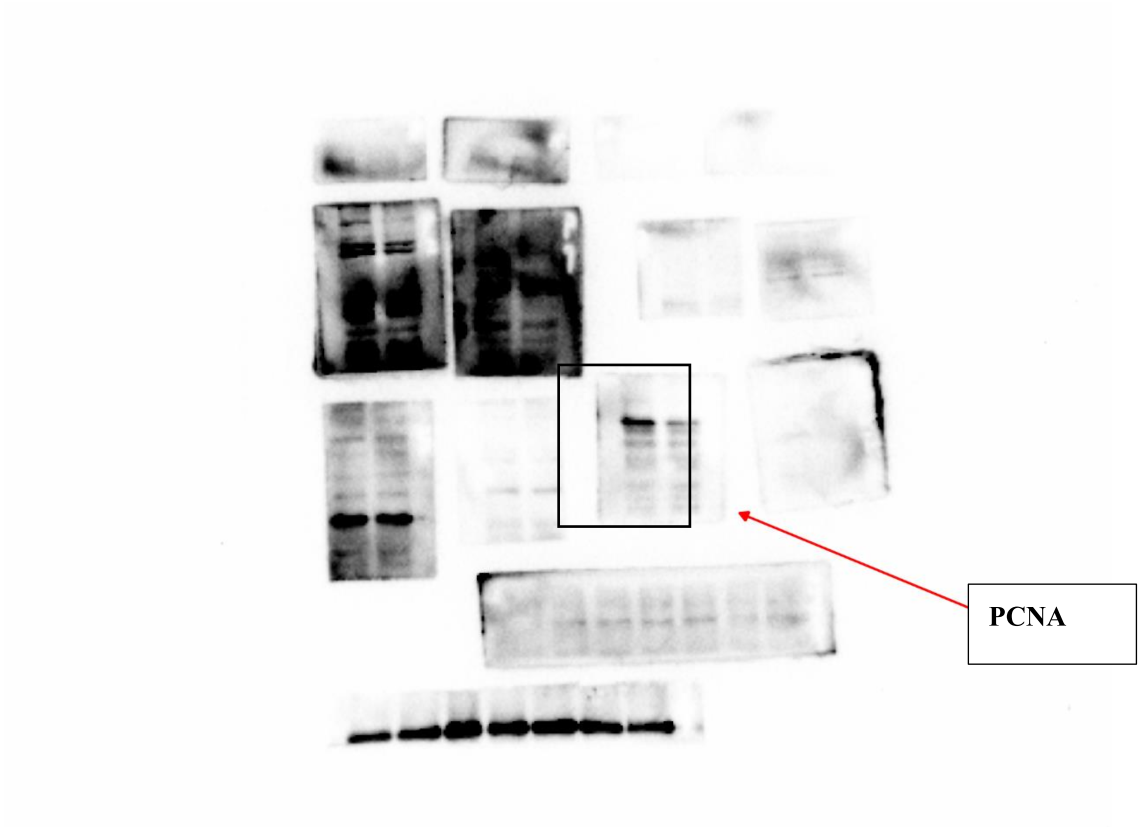

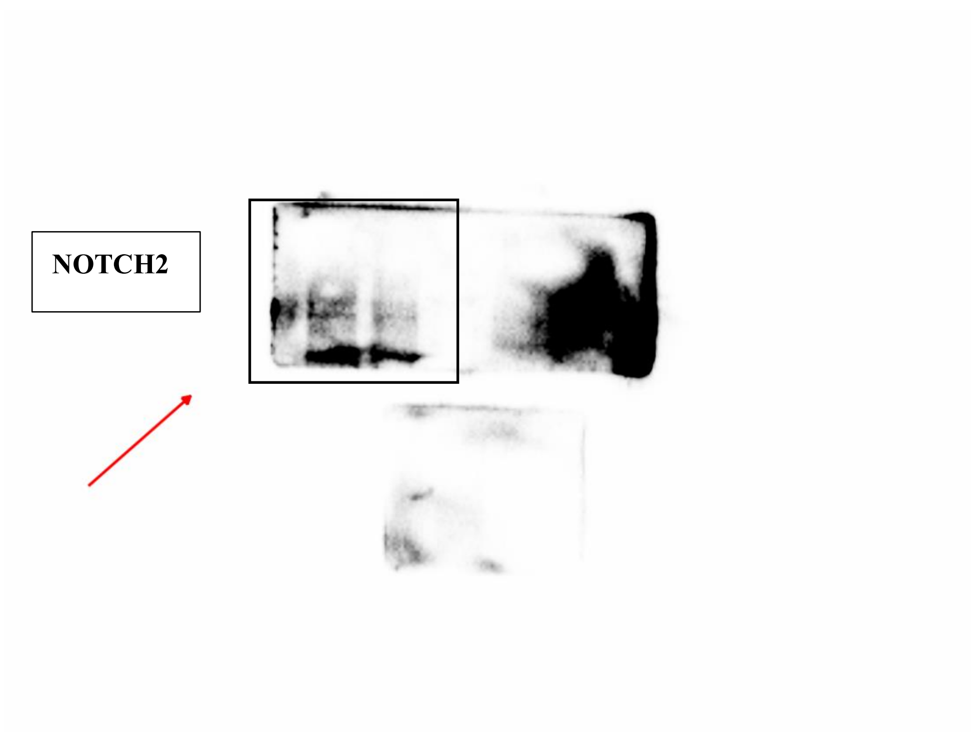
NOTCH2

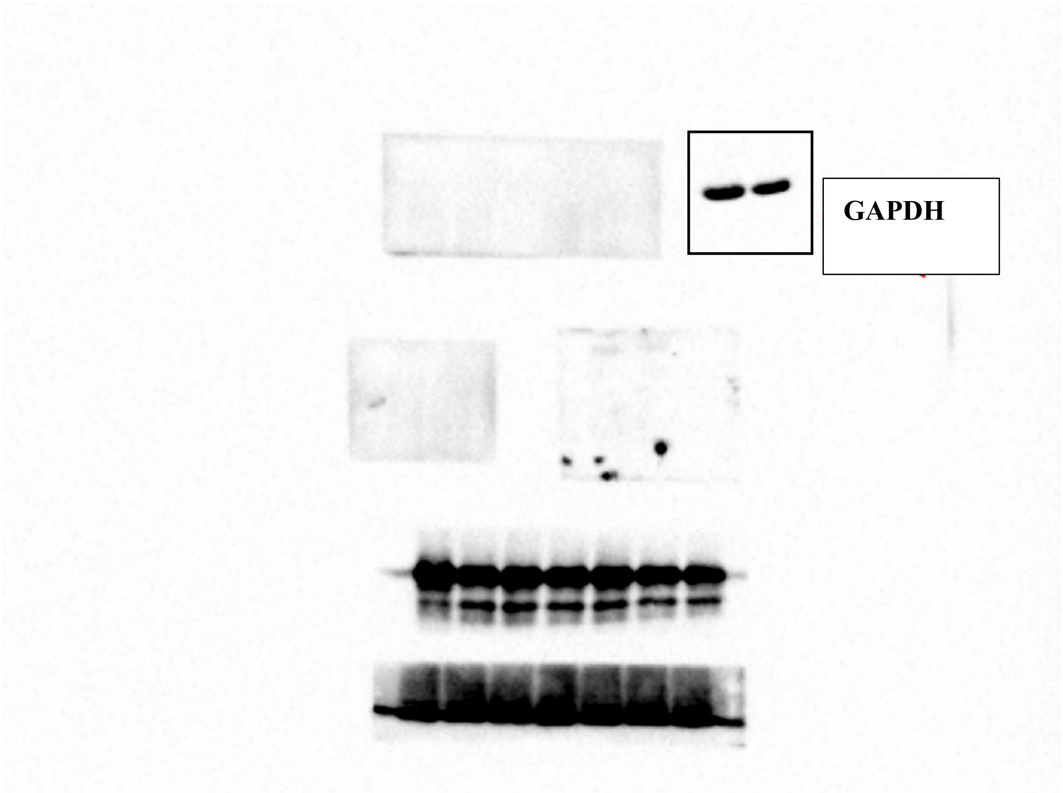
GAPDH

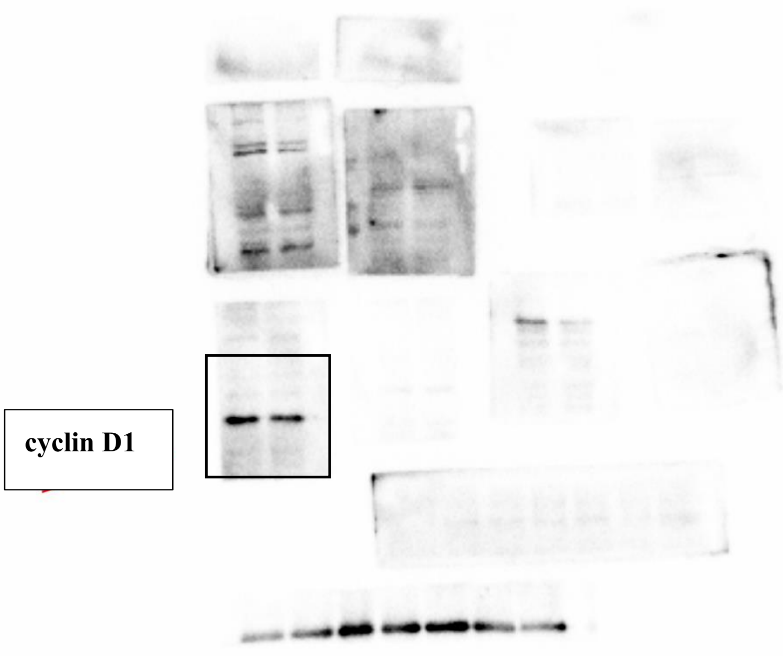
cyclin D1

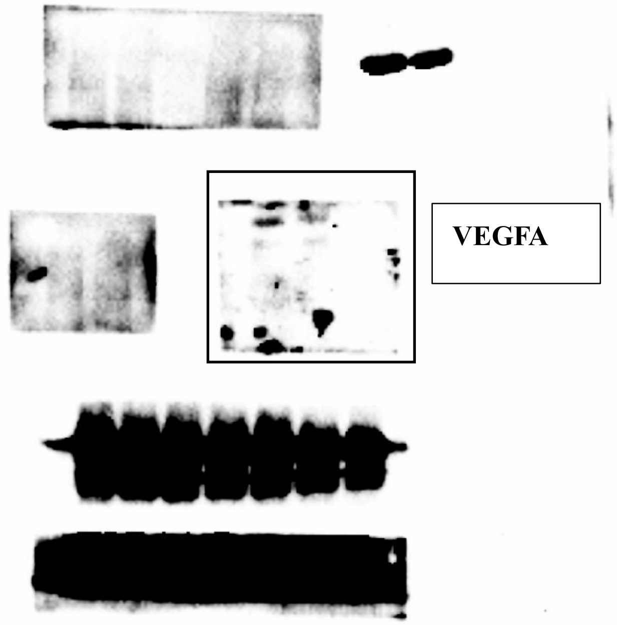
VEGFA
